# Supplementary material for: Individual and contextual predictors of emergency department visits among community-living older adults: a register-based prospective cohort study
Source: BMJ Open. 2022 Feb 9;12(2):e055484. doi: 10.1136/bmjopen-2021-055484 (PMC8830250; doi:10.1136/bmjopen-2021-055484)
Supplement: Author's manuscript [file bmjopen-2021-055484.draft_revisions.pdf]

BMJ Open is committed to open peer review. As part of this commitment we make the peer review history of every article we publish publicly available.

When an article is published we post the peer reviewers' comments and the authors' responses online. We also post the versions of the paper that were used during peer review. These are the versions that the peer review comments apply to.

The versions of the paper that follow are the versions that were submitted during the peer review process. They are not the versions of record or the final published versions. They should not be cited or distributed as the published version of this manuscript.

BMJ Open is an open access journal and the full, final, typeset and author-corrected version of record of the manuscript is available on our site with no access controls, subscription charges or pay-per-view fees (http://bmjopen.bmj.com).

If you have any questions on BMJ Open's open peer review process please email info.bmjopen@bmj.com

BMJ Open

Individual and contextual predictors of emergency department visits among community-living older adults: A register-based prospective cohort study

Journal:	*BMJ Open*
Manuscript ID	bmjopen-2021-055484
Article Type:	Original research
Date Submitted by the Author:	13-Jul-2021
Complete List of Authors:	Naseer, Mahwish; Dalarna University, School of Health and Welfare; Karolinska Institutet, Aging Research Center, Department of Neurobiology, Care Sciences and Society<br>McKee, Kevin; Dalarna University, School of Health and Welfare<br>Ehrenberg, Anna ; Dalarna University, School of Health and Welfare<br>Schön, Pär; Aging Research Center<br>Dahlberg, Lena; Dalarna University, School of Health and Welfare; Karolinska Institutet & Stockholm University, Aging Research Center
Keywords:	PUBLIC HEALTH, International health services < HEALTH SERVICES ADMINISTRATION & MANAGEMENT, ACCIDENT & EMERGENCY MEDICINE

SCHOLARONE™
Manuscripts

I, the Submitting Author has the right to grant and does grant on behalf of all authors of the Work (as defined in the below author licence), an exclusive licence and/or a non-exclusive licence for contributions from authors who are: i) UK Crown employees; ii) where BMJ has agreed a CC-BY licence shall apply, and/or iii) in accordance with the terms applicable for US Federal Government officers or employees acting as part of their official duties; on a worldwide, perpetual, irrevocable, royalty-free basis to BMJ Publishing Group Ltd ("BMJ") its licensees and where the relevant Journal is co-owned by BMJ to the co-owners of the Journal, to publish the Work in this journal and any other BMJ products and to exploit all rights, as set out in our licence.

The Submitting Author accepts and understands that any supply made under these terms is made by BMJ to the Submitting Author unless you are acting as an employee on behalf of your employer or a postgraduate student of an affiliated institution which is paying any applicable article publishing charge ("APC") for Open Access articles. Where the Submitting Author wishes to make the Work available on an Open Access basis (and intends to pay the relevant APC), the terms of reuse of such Open Access shall be governed by a Creative Commons licence – details of these licences and which Creative Commons licence will apply to this Work are set out in our licence referred to above.

Other than as permitted in any relevant BMJ Author's Self Archiving Policies, I confirm this Work has not been accepted for publication elsewhere, is not being considered for publication elsewhere and does not duplicate material already published. I confirm all authors consent to publication of this Work and authorise the granting of this licence.

For peer review only

TITLE PAGE

Individual and contextual predictors of emergency department visits among community-living older adults: A register-based prospective cohort study

Running title: Predictors of emergency department visits

Mahwish Naseer[a,b] MSc, MPH, Kevin J. McKee[a] PhD, Anna Ehrenberg[a] PhD, Pär Schön[b] PhD, Lena Dahlberg[a,b] PhD

[a]School of Health and Welfare, Dalarna University, SE-79188 Falun, Sweden

[b]Aging Research Center, Karolinska Institutet & Stockholm University, Tomtebodavägen 18A, SE-17165 Solna, Sweden

Corresponding author

Mahwish Naseer

School of Health and Welfare, Dalarna University,

SE-79188 Falun, Sweden

mna@du.se

Word counts:

Abstract 240

Manuscript (excluding abstract, summary, and references) 3522

Number of tables 5

For peer review only

Abstract

Objectives: To examine the extent to which contextual factors explain ED visits and ED revisits, additional to that explained by individual factors.

Design: A register-based prospective cohort study.

Setting: Swedish region of Dalarna.

Participants: The study sample was community-living adults aged 80 or older, resident in a region of Sweden, excluding older adults who moved out of Dalarna or into residential care during the study period. The analytical sample was N =16,543.

Outcome measures: Dependent variables were initial ED visit, and at least one ED revisit within 30 days of an initial ED visit.

Results: Approximately 36% of the sample visited the ED, of whom 19% had an ED revisit. For both ED visits and ED revisits, the addition of contextual factors to models containing individual factors significantly improved model fit (p<.001; p<.022) and the amount of variance explained in the outcome. In the final models, ED visits was significantly associated with older age, number of chronic diseases, receipt of home help, number of primary care visits, proportion of 80+ in the population and shorter distance to the ED; while ED revisits was significantly associated with greater use of social care, number of hospital admissions, and disposition at initial ED visit.

Conclusion: Contextual factors explain variance in ED visits, additional to that explained by individual factors alone, which indicates inequitable access to ED care. These findings suggest considering local variations in order to improve health related outcomes among older adults.

Key words: health care use; acute care; home help use; Andersen model; older people

For peer review only

Strengths and limitations of this study

- Contextual factors are included in this study in seeking to explain ED visits which have been rarely considered previously.
- This is a register-based study and registers provide high quality information on health status and health and social care use covering the entire population.
- Levels and quality of home help receipt were used as factors in ED visits rather than simply use/non-use.
- Administrative differences in how municipalities report data to the database may impact data reliability of municipal level data.

For peer review only

1. Introduction

Emergency department (ED) visits are increasing worldwide, resulting in crowding and concern about the appropriateness of such visits.[1] A systematic review on ED visits found that older adults accounted for 12-21% of all ED visits.[2] In Sweden, adults aged 80 years or older constitute 5% of the total population yet account for 18% of all ED visits.[3] Older adults' complex health needs are associated with a relatively high use of health services but do not necessarily imply a requirement of ED care,[2] which is often poorly adapted to such needs and may carry a risk of negative outcomes.[4,5] While EDs provide treatment for health problems and injuries that require immediate attention, some ED visits are partly explained by an unmet need for primary and social care for older adults[2,6] The well-established Andersen model of health service use proposes the consideration of contextual factors along with individual factors for a better understanding of health care use.[7] This study explores whether considering contextual factors in addition to individual factors can improve our understanding of ED visits among older adults.

Individual factors such as age, gender and health problems could potentially explain the use of different care services. There are mixed findings on the association between old age and ED visits, [8-10] possibly due to inconsistent methodology (e.g., definition of old age, variation in setting and outcome measures). There are higher odds of primary care use among women, and ED and inpatient hospital care use among men.[8,11,12] Primary and social care play an important role in managing chronic diseases and in the proactive treatment of health problems that might require an ED visit.[2] However, research has shown that home help receipt and greater use of primary care are associated with ED revisits and unplanned hospitalisations.[11, 13] While it is appropriate that greater need for care should be associated with greater use of services, service use does not necessarily ensure that care needs are met.[14] Low staff competence and continuity entails a risk of unmet needs and may lead to avoidable ED visits.

For peer review only - http://bmjopen.bmj.com/site/about/guidelines.xhtml

In Sweden, the proportion of registered nurses in municipal social care for older adults is lower than in neighbouring Norway (9% vs 31%),[15] while currently a person receiving home help meets an average of 16 different carers fortnightly.[3] However, studies on health care use have focused on home help receipt *per se*, rather than its level (instrumental services, personal care or both) and quality .[11, 13, 16]

Contextual factors related to health care use include community characteristics such as the population age distribution, geographical location, and the organisation of health and social care.[7] In Sweden, health and social care is financed, managed and provided by 21 regions and 290 municipalities, with local variations in the management and delivery of care services. For example, without national guidelines on eligibility, municipalities decide on eligibility criteria for services provided via home help and residential care.[17] This challenges the idea that all citizens in a universal welfare state should have equitable access to high quality care services driven by their need rather than, e.g., place of residence.[18, 19]

Health and social care systems internationally have undergone substantial changes. For example, Sweden has the lowest per capita hospital bed rate in Europe,[20] contributing to shorter lengths of hospital stay and an increased need of primary care, and a 30 percent reduction in residential care since 2000.[17] A growing proportion of older adults are thus ageing at home and the availability of home help services is key to their living independently.[21] However, home help provision has not fully compensated for the decrease in residential care.[22] Instead, there has been an increase in informal care, particularly for older adults with lower education, while those with higher education are more likely to buy private services.[18]

Contextual factors potentially influencing ED care are largely unexplored. A French study found that shorter distance to the ED and unavailability of primary care are associated with ED visits.[23] Studies on social care supply for older adults have shown that higher social care

For peer review only - http://bmjopen.bmj.com/site/about/guidelines.xhtml

expenditures and greater availability of residential care are negatively associated with ED revisits.[21, 24] However, there are few studies on the association between home help coverage and ED visits.[21]

In summary, while both individual and contextual factors have the potential to explain ED care use in older adults, research has primarily focused on individual factors. Moreover, few studies on ED visits and revisits have considered the level and quality of home help receipt.

The aim of this study is to examine the extent to which contextual factors can improve the explanation of ED visits and ED revisits by community-living older adults, compared to that provided by individual factors alone.

2. Methods

2.1 Study design, setting and population

A prospective cohort design was used. The study population was community-living adults aged 80 or older, resident in the Swedish region of Dalarna on 31st December 2014 (N=17,077). Excluding older adults who moved out of Dalarna (N=65) or into residential care during 2015 (N=469), the analytical sample was N =16,543.

There are fifteen municipalities in Dalarna whose population densities vary from 1.0 to 90.1 persons/km^2. Thus, great geographical variation is expected in health care and social care access. All hospital EDs are 24-hour operational, one regional (Falun) and three local (Mora, Avesta, and Säter; Säter is for psychiatric care patients only).

2.2 Patient and Public Involvement

Patients or the public were not involved in the design or conduct of study.

2.3 Data sources

For peer review only - http://bmjopen.bmj.com/site/about/guidelines.xhtml

This study is based on data from three registers: two national (the Longitudinal Integration Database for Health Insurance and Labour Market (LISA) and the Social Services Register) and one regional (the health care database of Region Dalarna), all of which cover their respective entire populations. The registers were linked via encrypted personal numbers that are assigned to all individuals living in Sweden. Municipal-level data was accessed from the publicly available database Kolada (https://www.kolada.se/), which is managed by the Council for the Promotion of Municipal Analyses.

Ethical approval for the study was awarded by the Regional Ethical Review Board in Stockholm (reg.no. 2016/299-31).

2.4 Dependent variables (DVs)

There were two dependent variables: i) *initial ED visit* during 2015, and ii) at least one *ED revisit* within 30 days of an initial ED visit (both coded as no (0); yes (1)).[9, 11]. Information on ED visits was obtained from the regional database.

2.5 Independent variables (IVs)

Individual factors

Individual factors were categorised into two domains: a) demographic and health characteristics, and b) home help receipt and health care use.

Demographics and health: Information on *age, gender*, and municipality of residence was obtained from LISA. Information on registered diagnosis (based on ICD-10 classification) at inpatient and outpatient visits during 2014 was obtained from the regional database and converted using a validated measure of chronic multi-morbidity in older adults into *number of chronic diseases*.[25]

For peer review only - http://bmjopen.bmj.com/site/about/guidelines.xhtml

Home help receipt and health care use: Information on *home help receipt* status at ED visit (or status in December 2014 for those who did not visit the ED during the study period) was obtained from the Social Services Register. Home help receipt was categorised as no home help, only instrumental services (e.g., cleaning, shopping), only personal care (e.g., hygiene), and both instrumental services and personal care.

Information on number of *primary care visits*, *specialist care visits*, and *hospital admissions* 12 months prior to the initial ED visit (or during 2014 for those who did not visit the ED during the study period) and *disposition at ED visit* (discharged; admitted to hospital) was obtained from the regional database, the latter IV only analysed in relation to the ED revisit DV.

Contextual factors

Contextual factors covered municipality-level data on: the *proportion of persons aged 80+ years* in the total population; annual *social care expenditures* per person aged 80+ years; *home help quality*; *median days in residential care* for adults aged 65+ resident in long-term care facilites; and *distance to the ED*. Distance to the ED which was estimated via Google maps and measured in kilometers as the shortest route from the centre of the municipality of residence to the nearest ED.[23] Since the ED in Säter is only for psychiatric care it was excluded in this estimation.

Two indicators were used of home help quality, based on data from an annual national survey distributed to adults aged 65+ years receiving home help and residential care.[26] Each indicator comprises the proportion of individuals within each municipality who provided only positive responses to questions on home help quality, with three questions in each of two domains: 1) *response, trust, and safety* and 2) *influence and adequate time*.

2.6 Data analysis

For peer review only - http://bmjopen.bmj.com/site/about/guidelines.xhtml

SPSS version 27 for Windows was used to conduct all analyses. Descriptive analyses as appropriate were performed for all individual factors for the total sample and within municipalities, and for contextual factors at the municipality level. Odds ratios with 95% confidence intervals (CI) were calculated for the bivariate associations between individual and contextual factors (IVs) and ED visits and ED revisits (DVs).

Subsequently, all IVs with a significant bivariate association with the DVs were used to develop multivariable models of the DVs. First, since the data for ED visits and ED revisits were nested within municipalities, the relevance of nested models was tested by Generalized Linear Mixed Models.[27] The variance estimate at municipality level was 0.064 for initial ED visit and 0.019 for revisit, providing an intraclass coefficient (ICC) of 0.019 and 0.005. Thus, only 1.9% of the variance in initial visit and 0.57% in revisit was explained by municipality of residence, that is, considerably below the conventional level indicating a multilevel analysis is required.[28] Therefore, we proceeded with sequential logistic regression.

Model building proceeded in three steps. At the first step, individual demographic and health characteristics were entered in the models. At the second step, individual home help receipt and health care use were entered in the models and changes in model fit statistics examined. At the third step, contextual factors were entered in the models to determine if there was significant improvement in the models after entry of all individual factors. The deviance statistic (-2LL), Cox and Snell's R^2_{CS} and Nagelkerke R^2_N test were used for model fitting.[29] The deviance describes the unexplained variance in the model, so the smaller the value of deviance, the better the model fits the data. R^2_{CS} and R^2_N are approximations to the R^2 statistic for multiple linear regression which describes the variance in the DV explained by the model. R^2_{CS} and R^2_N are calculated differently and may provide divergent estimates, thus both were included.[29]

For peer review only - http://bmjopen.bmj.com/site/about/guidelines.xhtml

Variance inflation factor (VIF) was used to check potential multicollinearity among independent variables. VIF values were below 10 for all the independent variables and considered acceptable.[30]

3. Results

3.1 Descriptive analyses

The mean age in the sample was 85.2 years and 58.5% were women (Table 1). The mean number of chronic diseases was 2.1 (range 1.8-2.4 across municipalities). Seventy-four percent lived at home with no home help, while 5.1% received instrumental services only, 3.9% personal care only, and 16.3% received instrumental services and personal care (range for home help receipt 19.1%-37.1%).

The mean number of primary care visits was 9.0 (range 7.5-10.4), while the mean number of specialist care visits was 2.7 (range 1.7-4.1) (Table 2). The mean number of hospital admissions was 0.3 (range 0.3-0.4). In the sample, 36.4% had at least one ED visit, of whom 18.9% had an ED revisit within 30 days. The mean number of ED visits was 0.7 (range 0.4-0.9).

Contextual factors are presented in Table 3. The proportion of adults aged 80+ years varied across municipalities from 5.3% to 9.0%, while social care costs per person varied from SEK 207,741 to SEK 272,317 (approx. USD 25,000-32,000). Positive home help quality varied between 35.0 % and 56.0% for response, trust, and safety and between 34.0% and 60.0% for influence and adequate time. Median days in residential care varied between 407 and 867, while distance to the nearest ED varied from 1.8 km to 78.8 km.

3.2 Initial ED visit

For peer review only - http://bmjopen.bmj.com/site/about/guidelines.xhtml

In bivariate analyses, individual factors significantly positively associated with initial ED visit were: age, number of chronic diseases, home help receipt, number of primary care visits, specialist care visits, and hospital admissions (Table 4). Contextual factors significantly negatively associated with ED visits were: median days in residential care, proportion of adults 80+ years in the population and greater distance to the ED.

In model 1 with the demographic and health characteristics age and number of chronic conditions included, deviance=20624.7, R^2_{CS}=.062 and R^2_N=.085. Compared to a constant-only model, model 1 is a significant improvement ($\chi^2(2)$=1065.2, p<.001). In model 2 with the addition of variables measuring home help and health care use, deviance=19883.9, R^2_{CS}=.103 and R^2_N=.142. Compared to model 1, model 2 is a significant improvement (model $\chi^2(8)$=1806.1, p<.001; step $\chi^2(6)$=740.9, p<.001). In model 3 with the addition of variables measuring contextual factors, deviance=19701.7, R^2_{CS}=.113 and R^2_N=.155. Compared to model 2, model 3 is a significant improvement (model $\chi^2(11)$=1988.3, p<.001; step $\chi^2(3)$=182.2, p<.001. Thus, the multivariable models indicated a better fit at each step.

In the final multivariable model (model 3), individual level factors with significant higher odds ratios for ED visits were: age, number of chronic diseases, home help receipt, and number of primary care visits. Contextual factors significantly associated with ED visit were: higher proportion of adults 80+ years in the total population and shorter distance to the ED.

3.3 ED revisit

In bivariate analyses, number of chronic diseases, receipt of both instrumental and personal care from home help services, number of primary care visits and hospital admissions prior to initial ED visit were significantly positively associated with ED revisit, while being admitted to hospital at initial ED visit was significantly negatively associated with an ED revisit. (Table

For peer review only - http://bmjopen.bmj.com/site/about/guidelines.xhtml

5). Of the contextual factors, distance to the ED was significantly negatively associated with an ED revisit.

In model 1 with the health characteristic number of chronic conditions included, deviance=5718.9, R^2_{CS}=.004 and R^2_N=.006. Compared to a constant-only model, model 1 is a significant improvement ($\chi^2(1)$=22.4, p<.001). In model 2 with the addition of variables measuring home help and health care use, deviance=5650.0, R^2_{CS}=.015 and R^2_N=.025. Compared to model 1, model 2 is a significant improvement (model $\chi^2(7)$=91.3, p<.001; step $\chi^2(6)$=68.9, p<.001). In model 3 with the addition of the contextual factor distance to the nearest ED, deviance=5644.8, R^2_{CS}=.016 and R^2_N=.026. Compared to model 2, model 3 is a significant improvement (model $\chi^2(8)$=96, p<.001; step $\chi^2(1)$=5.26, p<.022). Thus, the multivariable models indicated a better fit at each step.

In the final multivariable model (model 3), receipt of both instrumental and personal care from home help services, number of hospital admissions, and disposition at initial ED visit were significantly associated with ED revisits. Although the addition of distance to nearest ED in model 3 resulted in a significant improvement in model fit compared to model 2, as the CIs of the odds ratio for the variable contains 1 we conclude that the association between distance to ED and ED revisit was not significant in the model.

4. Discussion

Approximately 36% of the sample visited the ED, of whom 19% had an ED revisit within 30 days. Research has mainly focused on individual predictors of ED care. Our study indicates that ED care can be further explained by contextual factors. Contextual factors associated with higher odds of initial ED visit indicate inequitable access to ED care.

Our findings on the associations between age, number of chronic diseases, and initial ED visits are in line with Andersen's model, which describes equitable access to ED care

For peer review only

For peer review only - http://bmjopen.bmj.com/site/about/guidelines.xhtml

explained by demographic factors (e.g., age) and need (e.g., chronic diseases).[7] Health decline with age relates to the increasing need for health care. Grunier et al. (2011) argued that proactive care can play a role in the management of need and can contribute positively to reducing ED visits. Our study shows that the addition of home help and health care variables increased the variance explained in DVs by demographics and health characteristics and improved the overall model fit. Positive associations between initial ED visit and both home help receipt and primary care visits suggest that the provided services do not meet the recipient's care needs [2], that is, indicate a poorly functioning health and social care system. However, extensive home help provision is also an indication of poor health/functional status with an increased risk for ED visits.

The needs of older adults are often complex, and inadequate co-ordination between hospitals, primary and municipal social care for older adults increases the risk of negative health outcomes. In Sweden, as in other high-income countries, the fragmentation of health and social care systems has become more visible during the COVID-19 pandemic, and there is a need to establish channels that ensure continuous and rapid coordination of services from different care providers and levels of organisations.[31, 32] Research has shown that integrated care can reduce ED visits, hospital admissions, and the length of hospital stay.[33, 34]

The ED visit model explains more variance in the outcome than the revisit model and more IVs are significant in the model, including contextual factors. This may be partly explained by the revisit model having less variation in its DV, and perhaps also due to ED revisits more than initial ED visit being related to extraneous variables that were not available in the registers accessed and thus not included in the models. The odds of revisits were higher in those who were not admitted to inpatient hospital care at initial visit and among those with greater use of home help services and higher number of hospital admissions. With shorter lengths of hospital stay,[20] municipalities delivering home help become responsible for older

For peer review only - http://bmjopen.bmj.com/site/about/guidelines.xhtml

adults with more complex care needs.[3] A recent report showed that patients who were sent home after initial ED visit had a declining health status at ED revisit within 30 days, i.e., that their needs for aftercare were not met.[35]

Our results show that contextual factors (living in municipalities with higher proportions of adults aged 80+ in the population and a shorter distance to the ED) increased the odds of an initial ED visit at the individual level. Access to ED care should be driven by need rather than place of residence, and the variance in initial ED visit explained by contextual factors challenges the idea of universalism and equitable access to health care.

Strengths and limitations

A strength of this study was that it was based on register data. Registers provide high quality information on health status and health and social care use covering the entire population. This study also included contextual factors in seeking to explain ED visits, which have been rarely considered previously, as well as levels and quality of home help receipt as factors in ED visits rather than simply use/non-use.

Home help receipt was categorised as an individual factor, while home help quality was categorised as a contextual variable as the individual-level data was only available for analysis as aggregated data. Thus, some variables that might explain ED visits or revisits can be optionally conceptualised and operationalised at individual or contextual levels. An association between variables can differ in strength and direction when analysed at individual versus group level, so it is important that the use of aggregated data as a contextual factor can be defended. The reliability of the data on home help quality will have been influenced by a survey response rate that varied between 48-66% by municipality and only 46% of the data provided by care recipients with the rest provided by relatives or care providers.

For peer review only - http://bmjopen.bmj.com/site/about/guidelines.xhtml

Patients who were admitted to the hospital compared to those discharged after the initial ED visit did not have an equal chance to be included within the 30 days criterion for an ED revisit. This problem is minor, though, since the median length of stay in hospital was 4 days. Administrative differences in how municipalities report data to the Kolada database may impact data reliability. Finally, distance to the nearest ED was estimated from the city centre rather than place of residence.

For peer review only

5. Conclusion

The aim of this study was to consider whether contextual factors improve our understanding of ED visits. Results suggest that contextual factors explain additional significant variance in initial ED visit compared to that explained by individual factors alone, which indicates inequitable access to ED care.

In individual factors, age and health characteristics explain variance in initial visit while receipt of home help and health care use explained variance both in initial visit and revisit. ED revisit was also explained by discharge from ED and can be interpreted as a result of inadequate care and discharge planning at the initial visit. Greater use of home help and primary care does not ensure that needs are met, and further research on the impact of unmet care needs in older adults on ED visits can clarify how the use of different health and social care services are associated. Local variations in home help receipt and utilisation of ED care challenge the notion of a universal welfare state, and require consideration in order to improve health related outcomes among older adults.

Funding: The study was supported by Swedish Research Council for Health, Working Life and Welfare (grant no. 2015-00440) and Region Dalarna. The funding organisation had no role in the development of study design, analysis, and/or in manuscript writing.

For peer review only - http://bmjopen.bmj.com/site/about/guidelines.xhtml

Conflict of Interest: The authors declare that they have no conflict of interest.

Author Contribution: All authors were involved in the development of study design, interpreting analyses, and results, and revising and finalising the manuscript. MN conducted data analysis, drafted and finalised the manuscript with inputs from all authors. KM provided advice regarding data analysis and support in the writing of the results section. All authors read and approved the final version of the manuscript.

Patient consent: Not applicable

Data availability statement: Data may be obtained from a third party and are not publicly available.

References

1. Boyle A, Beniuk K, Higginson I, Atkinson P. Emergency department crowding: time for interventions and policy evaluations. Emerg Med Int. 2012; 2012:838610.

2. Gruneir A, Silver MJ, Rochon PA. Emergency department use by older adults: A literature review on trends, appropriateness, and consequences of unmet health care needs. Med Care Res Rev 2011; 68(2): 131-155.

3. National Board of Health and Welfare. Vård och omsorg om äldre lägesrapport 2020 [The national report on social care for older adults 2020]. Stockholm: National Board of Health and Welfare.

4. Covinsky KE, Pierluissi E, Johnston CB. Hospitalization-associated disability: "She was probably able to ambulate, but I'm not sure". JAMA 2011; 306(16): 1782–1793.

5. SBU. Omhändertagande av äldre som inkommer akut till sjukhus-med fokus på sköra äldre. En systematisk litteratur översikt [Care of older people coming to hospital emergency: a focus

For peer review only - http://bmjopen.bmj.com/site/about/guidelines.xhtml

on frail older people. A systematic literature review] 2013. Stockholm: Swedish Agency for Health Technology Assessment.

6. Hass Z, DePalma G, Craig BA, Xu H, Sands LP. Unmet need for help with activities of daily living disabilities and emergency department admissions among older medicare recipients. Gerontologist 2017; 57(2): 206-210.

7. Andersen RM. National Health Surveys and the Behavioral Model of Health Services Use. Med Care 2008; 46(7): 647-653.

8.de Gelder J, Lucke JA, de Groot B et al. Predictors and outcomes of revisits in older adults discharged from the emergency department. J Am Geriatr Soc 2018; 66(4): 735-741.

9. Lowthian J, Straney LD, Brand CA et al. Unplanned early return to the emergency department by older patients: the Safe Elderly Emergency Department Discharge (SEED) project. Age Ageing 2016; 45(2): 255-261.

10.Lynch B, Fitzgerald AP, Corcoran P, Buckley C, Healy O, Browne J. Drivers of potentially avoidable emergency admissions in Ireland: an ecological analysis. BMJ Qual Saf 2019; 28(6): 438-448.

11. Naseer M, Agerholm J, Fastbom J, Schön P, Ehrenberg A, Dahlberg L. Factors associated with emergency department revisits among older adults in two Swedish regions: A prospective cohort study. Arch Gerontol Geriatr 2020; 86: 103960.

12. Friberg OI, Krantz G, Määttä S, Järbrink K. (2016). Sex differences in health care consumption in Sweden: A register-based cross-sectional study. Scand J Public Health 2016; 44(3); 264-273.

For peer review only - http://bmjopen.bmj.com/site/about/guidelines.xhtml

13. Dahlberg L, Agahi N, Schön P, Lennartsson C. Planned and unplanned hospital admissions and their relationship with social factors: findings from a national, prospective study of people aged 76 years or older. Health Ser Res 2018; 53(6): 4248-4267.

14. Cunningham A, Mautner D, Ku B, Scott K, LaNoue M. Frequent emergency department visitors are frequent primary care visitors and report unmet primary care needs. J Eval Clin Pract 2017; 23(3): 567-573.

15. SOU. Internationella erfarenheter av Covid-19 i äldreboende [International experiences of Covid-19 in residential care] 2020. Reports SOU Stockholm 2020: 80.

16. Tanderup A, Ryg J, Rosholm JU, Lassen AT. Association between the level of municipality healthcare services and outcome among acutely older patients in the emergency department: a Danish population-based cohort study. BMJ Open 2019; 9(4): e026881.

17. Schön, P., & Heap, J. European Social Policy Network (ESPN) Thematic report on challenges in long-term care Sweden 2018. Available at: https://ec.europa.eu/social/main.jsp?pager.offset=30&catId=792&langId=en&moreDocuments=yes. Accessed October 12, 2020.

18. Szebehely M, Trydegård GB. Home care for older people in Sweden: a universal model in transition. Health Soc Care Community 2012; 20(3): 300-309.

19. Trydegård GB, Thorslund M. One uniform welfare state or a multitude of welfare municipalities? The evolution of local variation in Swedish elder care. Soc Policy Adm 2010; 44(4); 495-511.

20. OECD. Health at a glance: Europe 2020. State of Health in the EU Cycle. OECD Publishing.

For peer review only - http://bmjopen.bmj.com/site/about/guidelines.xhtml

21. Spiers G, Matthews FE, Moffatt S, et al. Impact of social care supply on healthcare utilisation by older adults: a systematic review and meta-analysis. Age Ageing 2018; 48(1); 57-66.

22. Ulmanen P, Szebehely M. From the state to the family or to the market? Consequences of reduced residential eldercare in Sweden. Int J Soc Welfare 2015; 24: 81-92.

23. Or Z, Penneau A. A Multilevel Analysis of the determinants of emergency care visits by the elderly in France. Health Policy 2018; 122(8): 908-914.

24. Fernandez JL, Forder J. Consequences of local variations in social care on the performance of the acute health care sector. Appl Econ 2008; 40(12): 1503-1518.

25. Calderón-Larrañaga A, Vetrano DL, Onder G et al. Assessing and measuring chronic multimorbidity in the older population: A proposal for its operationalization. J Gerontol A Biol Sci Med Sci 2017; 72(10): 1417-1423.

26. National Board of Health and Welfare. Så tycker de äldre om äldreomsorgen 2015: En rikstäckande undersökning i hemtjänst och äldreboenden [What older people thinks on eldercare 2015: A national survey on home help and institutional care]. Stockholm: National Board of Health and Welfare.

27. Sommet N, Morseli D. Keep calm and learn multilevel logistic modelling: A simplified three-step procedures using Stata, R, Mplus, and SPSS. International Review Social Psychology 2017; 30 1(1): 203-218.

28. Kreft IGG, de Leeuw J. Introducing multilevel modeling, SAGE Publications, 1998.

29. Field A. Discovering statistics using IBM SPSS Statistics. 4th Ed. London: SAGE Publications, 2016.

For peer review only - http://bmjopen.bmj.com/site/about/guidelines.xhtml

30.Myers R. Classical and modern regression with applications. 1990. In: Field A. Discovering statistics using IBM SPSS Statistics. 4th Ed. London: SAGE Publications, 2016.

31. SOU. The elderly care in the pandemic 2020. Corona commission: Statens Offentliga Utredningar, Stockholm 2020 80. Available at: https://coronakommissionen.com/. Accessed January 30, 2021.

32. Estabrooks CA, Straus S, Flood, CM, et al. Restoring trust: COVID-19 and the future of long-term care. Royal Society of Canada. 2020. Available at: https://rsc-src.ca/en/themes/long-term-care. Accessed May 21st, 2021.

33. Liljas AEM, Brattström F, Burström B, Schön P, Agerholm J. Impact of integrated care on patient-related outcomes among older people-A systematic review. Int J Integr Care 2019; 19(3): 6, 1-6.

34. Dohney M, Agerholm J, Orsini N, Schön P, Burström B. Impact of integrated care on trends in the rate of emergency department visits among older persons in Stockholm county: an interrupted time series analysis. BMJ Open 2020; 10: e0361182.

35. Stockholm Gerontology Research Center. Äldres resa till, genom och ut från sjukhuset i Stockholms län: En lägesbeskrivning inför lagen om samverkan vid utskrivning från sluten hälso-och sjukvård [Older people’s way through hospital care in Region Stockholm: A discription of the situation before the implementation of the Act on coordinated discharge from hospital care] 2018. Reports Stockholm Gerontology Research Center 2018 1.

For peer review only - http://bmjopen.bmj.com/site/about/guidelines.xhtml

For peer review only

Table 1

Demographic, health and home help use characteristics for total study sample (N=16,543) and stratified by municipality

Municipality	n	Demographics		Health	Home help			
		Age	Female gender	No. of chronic diseases	No	Instrumental services	Personal care	Instrumental & personal care
		Mean (SD)		Mean (SD)	%	%	%	%
Avesta	1474	85.1 (4.1)	57.1	2.4 (2.0)	80.5	3.1	2.1	14.2
Borlänge	2599	85.1 (4.2)	57.5	2.0 (1.8)	72.0	1.3	12.	14.2
Falun	2925	85.2 (4.2)	58.5	2.2 (1.8)	62.9	14.0	1.1	22.0
Gagnef	539	85.1 (4.0)	54.2	2.1 (1.7)	79.8	2.0	4.0	14.1
Hedemora	932	85.1 (4.2)	59.5	2.2 (1.9)	78.8	2.3	2.0	16.8
Leksand	945	85.4 (4.2)	59.0	1.8 (1.7)	78.7	2.7	2.7	15.9
Ludvika	1732	85.5 (4.3)	60.9	2.1 (1.8)	75.6	6.6	2.1	15.6
Malung-Sälen	633	84.8 (4.1)	59.1	2.2 (1.9)	77.3	3.4	2.3	16.9
Mora	1215	85.4 (4.4)	59.6	2.0 (1.9)	78.2	2.2	2.8	16.7
Orsa	477	85.1 (4.1)	58.5	2.3 (2.0)	78.8	2.5	3.1	15.5
Rättvik	905	85.3 (4.1)	60.0	1.9 (1.8)	80.8	4.2	1.5	13.5
Smedjebacken	617	84.8 (4.0)	55.9	2.1 (1.8)	78.1	6.0	4.5	11.3

For peer review only - http://bmjopen.bmj.com/site/about/guidelines.xhtml

Säter	591	85.2 (4.2)	57.9	2.1 (1.8)	76.3	4.0	3.0	16.6
Vansbro	440	84.9 (4.0)	58.6	2.1 (1.9)	80.9	0.9	4.7	13.4
Älvdalen	519	85.0 (4.0)	59.0	2.2 (1.9)	75.7	4.2	3.6	16.4
Total	16,543	85.2 (4.2)	58.5	2.1 (1.8)	74.6	5.1	3.9	16.3

For peer review only

Table 2:

Health care use for total study sample (N=16,543) and stratified by municipality.

Municipality	n	No. of primary care visits	No. of specialist care visits	No. of hospital admissions	ED visits (Yes)	No. of ED visits	¤ED revisit
		Mean (SD)	Mean (SD)	Mean (SD)	%	Mean (SD)	%
Avesta	1474	7.5 (8.0)	3.0 (8.1)	0.4 (0.9)	38.9	0.8 (1.5)	23.5
Borlänge	2599	8.6 (9.3)	2.6 (8.5)	0.3 (0.8)	36.0	0.6 (1.1)	20.0
Falun	2925	10.4 (10.8)	2.9 (9.3)	0.3 (0.8)	41.8	0.8 (1.6)	19.1
Gagnef	539	8.4 (10.3)	2.2 (6.6)	0.3 (0.8)	34.0	0.6 (1.2)	21.3
Hedemora	932	8.1 (8.5)	2.5 (7.1)	0.3 (0.8)	36.8	0.7 (1.4)	22.3
Leksand	945	10.2 (10.9)	1.9 (3.7)	0.3 (0.8)	36.2	0.7 (1.3)	16.2
Ludvika	1732	8.8 (9.8)	3.0 (9.0)	0.3 (0.8)	26.2	0.4 (0.9)	15.6
Malung-Sälen	633	9.2 (9.0)	2.0 (7.4)	0.3 (0.7)	28.1	0.4 (0.9)	13.0
Mora	1215	9.2 (8.6)	3.3 (9.4)	0.4 (0.9)	44.7	0.9 (1.6)	17.8
Orsa	477	8.4 (8.7)	4.1 (16.8)	0.4 (0.8)	44.9	0.8 (1.4)	17.8
Rättvik	905	8.4 (9.0)	2.3 (6.4)	0.3 (0.8)	37.2	0.7 (1.2)	21.1
Älvdalen	519	8.5 (9.2)	2.6 (12.2)	0.3 (0.7)	38.2	0.6 (1.1)	17.6
Smedjebacken	617	8.9 (8.3)	2.2 (5.5)	0.3 (0.8)	26.1	0.4 (0.9)	15.0
Säter	591	9.2 (9.8)	1.7 (3.8)	0.3 (0.7)	33.3	0.5 (1.1)	13.9
Vansbro	440	9.8 (9.6)	2.2 (7.0)	0.3 (0.8)	31.4	0.5 (1.1)	18.4
Älvdalen	519	8.5 (9.2)	2.6 (12.2)	0.3 (0.7)	38.2	0.6 (1.1)	17.6
Total	16,543	9.0 (9.6)	2.7 (8.5)	0.3 (0.8)	36.4	0.7 (1.3)	18.9

Note: Abbreviations: ED, emergency department; SD, standard deviation

¤ Sample for ED revisit includes only those who visited ED (n = 5930).

For peer review only

For peer review only - http://bmjopen.bmj.com/site/about/guidelines.xhtml

Table 3

Contextual factors at municipality level

Municipality	Proportion of 80+ in the population	Annual social care cost per person 80+	Positive home help quality		Median number of days in residential care	Distance to the nearest ED
			Response, trust, & safety	Influence & adequate time		
	%	SEK	%	%		km
Avesta	6.7	207,741	41.0	44.0	578	2.3
Borlänge	5.3	229,601	37.0	41.0	585	21.3
Falun	5.4	244,487	48.0	60.0	573	1.8
Gagnef	5.7	255,825	44.0	57.0	610	40.2
Hedemora	6.3	218,694	40.0	53.0	680	20.9
Leksand	6.7	235,201	50.0	51.0	561	49.3
Ludvika	7.0	224,179	42.0	46.0	715	64.4
Malung-Sälen	7.0	223,591	51.0	55.0	867	73.0
Mora	6.4	250,077	40.0	46.0	407	2.00
Orsa	7.3	221,582	37.0	34.0	740	12.1
Rättvik	9.0	209,722	45.0	49.0	597	35.6
Smedjebacken	6.1	215,149	39.0	40.0	647	60.8
Säter	5.8	229,107	56.0	53.0	506	43.2
Vansbro	7.0	219,152	35.0	39.0	828	78.8
Älvdalen	7.5	272,317	52.0	49.0	711	41.7

For peer review only

For peer review only - http://bmjopen.bmj.com/site/about/guidelines.xhtml

Table 4

Models for binary logistic regression of factors associated with initial ED visit (N = 16,543)

	ED visit			
	Bivariate OR (95% CI)	Model 1 OR (95% CI)	Model 2 OR (95% CI)	Model 3 OR (95% CI)
Individual factors				
**Demographics & health**				
Age	1.08 (1.07-1.09)*	1.09 (1.08-1.09)*	1.06 (1.05-1.06)*	1.06 (1.05-1.07)*
Gender				
Female	Ref.			
Male	1.04 (0.97-1.11)			
No. of chronic diseases	1.22 (1.20-1.25)*	1.24 (1.21-1.26)*	1.12 (1.10-1.15)*	1.12 (1.10-1.15)*
**Home help & health care use**				
Home help				
No	Ref.		Ref.	Ref.
Instrumental services	1.74 (1.51-2.01)*		1.31 (1.13-1.52)*	1.26 (1.08-1.46)*
Personal care	2.00 (1.71-2.34)*		1.49 (1.26-1.76)*	1.55 (1.31-1.83)*
Instrumental & personal care	3.38 (3.10-3.68)*		2.49 (2.27-2.74)*	2.48 (2.26-2.73)*
No. of primary care visits	1.05 (1.04-1.05)*		1.03 (1.03-1.04)*	1.03 (1.03-1.04)*
No. of specialist care visits	1.02 (1.01-1.02)*		1.00 (1.00-1.01)	1.00 (1.00-1.01)
No. of hospital admissions	1.37 (1.32-1.42)*		1.01 (0.96-1.05)	1.00 (0.96-1.05)
Contextual factors				
Annual social care cost per person 80+	1.00 (1.00-1.00)			
Response, trust, & safety	1.00 (0.99-1.00)			
Influence & adequate time	1.00 (1.00-1.01)			
Median days in residential care	0.99 (0.99-0.99)*			1.00 (0.99-1.00)
Proportion of 80+ in the population	0.94 (0.91-0.98)*			1.09 (1.05-1.13)*
Distance to the nearest ED	0.99 (0.99-0.99)*			0.99 (0.98-0.99)*
Deviance (-2LL)		20624.749	19883.865	19701.645
Cox & Snell R^2_{CS}		0.062	0.103	0.113
Nagelkerke R^2_N		0.085	0.142	0.155

Note: The negative bivariate association for proportion of 80+ in the population with initial ED visit becomes a positive association and increases in magnitude in the multivariable model, indicating negative suppression. Through trial variable removal from the model, it was determined that distance to the ED and median days in residential care both acted as suppressor variables for proportion 80+ in the population.
Abbreviations: ED, emergency department; OR, odds ratio; CI, confidence interval
*Significant on values estimated from 95% CI

For peer review only

Table 5

Models for logistic regression of factors associated with ED revisit within 30 days after initial ED visit (n =5930)

	ED revisit			
	Bivariate OR (95% CI)	Model 1 OR (95% CI)	Model 2 OR (95% CI)	Model 3 OR (95% CI)
Individual factors				
**Demographics & health**				
Age	0.99 (0.98-1.01)			
Gender				
Female	Ref.			
Male	1.12 (0.98-1.27)			
No. of chronic diseases	1.07 (1.04-1.10)*	1.07 (1.04-1.10)*	1.02 (0.98-1.06)	1.02 (0.98-1.06)
**Home help & health care use**				
Home help				
No	Ref.		Ref.	Ref.
Instrumental services	0.99 (0.75-1.32)		0.99 (0.74-1.32)	0.97 (0.73-1.29)
Personal care	1.13 (0.84-1.53)		1.13 (0.84-1.53)	1.15 (0.85-1.55)
Instrumental & personal care	1.33 (1.15-1.55)*		1.31 (1.13-1.53)*	1.30 (1.12-1.51)*
No. of primary care visits	1.01 (1.01-1.02)*		1.00 (1.00-1.01)	1.00 (1.00-1.01)
No. of specialist care visits	1.00 (0.99-1.00)			
No. of hospital admissions	1.19 (1.13-1.26)*		1.15 (1.08-1.23)*	1.15 (1.08-1.23)*
Disposition at index ED visit				
Discharged	Ref.		Ref.	Ref.
Hospital admission	0.73 (0.65-0.83)*		0.69 (0.61-0.79)*	0.71 (0.62-0.81)*
Contextual factors				
Annual social care cost per person 80+	1.00 (1.00-1.00)			
Response, trust, & safety	0.98 (0.97-1.00)			
Influence & adequate time	0.99 (0.99-1.00)			
Median days in residential care	1.00 (0.99-1.00)			
Propotion of 80+ in the population	0.99 (0.92-1.06)			
Distance to the nearest ED	0.99 (0.99-0.99)*			0.99 (0.99-1.00)
Deviance (-2LL)		5718.922	5650.039	5644.775
Cox & Snell R^2_{CS}		0.004	0.015	0.016
NagelkerkeR^2_N		0.006	0.025	0.026

Note: Abbreviations: ED, emergency department; OR, odds ratio; CI, confidence interval

*Significant on values estimated from 95% CI

For peer review only

The RECORD statement – checklist of items, extended from the STROBE statement, that should be reported in observational studies using routinely collected health data.

	Item No.	STROBE items	Location in manuscript where items are reported	RECORD items	Location in manuscript where items are reported
**Title and abstract**					
	1	(a) Indicate the study's design with a commonly used term in the title or the abstract (b) Provide in the abstract an informative and balanced summary of what was done and what was found	a) Title and abstract (sub-heading: design)<br>b) Abstract (p.2)	RECORD 1.1: The type of data used should be specified in the title or abstract. When possible, the name of the databases used should be included.<br><br>RECORD 1.2: If applicable, the geographic region and timeframe within which the study took place should be reported in the title or abstract.<br><br>RECORD 1.3: If linkage between databases was conducted for the study, this should be clearly stated in the title or abstract.	Title and abstract (register)<br><br>Abstract<br><br>Linkage between databases was conducted for independent variables, therefore mentioned in the main text under subheading data sources (p. 7)
**Introduction**					
Background rationale	2	Explain the scientific background and rationale for the investigation being reported	p.4-6		
Objectives	3	State specific objectives, including any prespecified hypotheses	p.6		
**Methods**					

For peer review only

Study Design	4	Present key elements of study design early in the paper	p.6		
Setting	5	Describe the setting, locations, and relevant dates, including periods of recruitment, exposure, follow-up, and data collection	p.6		
Participants	6	*(a) Cohort study* - Give the eligibility criteria, and the sources and methods of selection of participants. Describe methods of follow-up<br>*Case-control study* - Give the eligibility criteria, and the sources and methods of case ascertainment and control selection. Give the rationale for the choice of cases and controls<br>*Cross-sectional study* - Give the eligibility criteria, and the sources and methods of selection of participants<br><br>*(b) Cohort study* - For matched studies, give matching criteria and number of exposed and unexposed<br>*Case-control study* - For matched studies, give matching criteria and the number of controls per case	p.6-7	RECORD 6.1: The methods of study population selection (such as codes or algorithms used to identify subjects) should be listed in detail. If this is not possible, an explanation should be provided.<br><br>RECORD 6.2: Any validation studies of the codes or algorithms used to select the population should be referenced. If validation was conducted for this study and not published elsewhere, detailed methods and results should be provided.<br><br>RECORD 6.3: If the study involved linkage of databases, consider use of a flow diagram or other graphical display to demonstrate the data linkage process, including the number of individuals with linked data at each stage.	N/A (register-based study covering entire population)<br><br>N/A<br><br>Register-based study covering entire population
Variables	7	Clearly define all outcomes, exposures, predictors, potential confounders, and effect modifiers. Give diagnostic criteria, if applicable.	p.7-8	RECORD 7.1: A complete list of codes and algorithms used to classify exposures, outcomes, confounders, and effect modifiers should be provided. If these cannot be reported, an explanation should be provided.	p.7-8
Data sources/ measurement	8	For each variable of interest, give sources of data and details	p.6-8		

For peer review only

		of methods of assessment (measurement).<br>Describe comparability of assessment methods if there is more than one group			
Bias	9	Describe any efforts to address potential sources of bias	N/A		
Study size	10	Explain how the study size was arrived at	N/A (entire population)		
Quantitative variables	11	Explain how quantitative variables were handled in the analyses. If applicable, describe which groupings were chosen, and why	p.7-8		
Statistical methods	12	(a) Describe all statistical methods, including those used to control for confounding<br>(b) Describe any methods used to examine subgroups and interactions<br>(c) Explain how missing data were addressed<br>(d) *Cohort study* - If applicable, explain how loss to follow-up was addressed<br>*Case-control study* - If applicable, explain how matching of cases and controls was addressed<br>*Cross-sectional study* - If applicable, describe analytical methods taking account of sampling strategy<br>(e) Describe any sensitivity analyses	p.8-9		
Data access and cleaning methods		..		RECORD 12.1: Authors should describe the extent to which the investigators had access to the database	p.6-7

For peer review only

				population used to create the study population.<br><br>RECORD 12.2: Authors should provide information on the data cleaning methods used in the study.	
Linkage		..		RECORD 12.3: State whether the study included person-level, institutional-level, or other data linkage across two or more databases. The methods of linkage and methods of linkage quality evaluation should be provided.	p.6-7
**Results**					
Participants	13	(a) Report the numbers of individuals at each stage of the study (*e.g.*, numbers potentially eligible, examined for eligibility, confirmed eligible, included in the study, completing follow-up, and analysed)<br>(b) Give reasons for non-participation at each stage.<br>(c) Consider use of a flow diagram	p.6	RECORD 13.1: Describe in detail the selection of the persons included in the study (*i.e.,* study population selection) including filtering based on data quality, data availability and linkage. The selection of included persons can be described in the text and/or by means of the study flow diagram.	p.6
Descriptive data	14	(a) Give characteristics of study participants (*e.g.*, demographic, clinical, social) and information on exposures and potential confounders<br>(b) Indicate the number of participants with missing data for each variable of interest<br>(c) *Cohort study* - summarise follow-up time (*e.g.*, average and total amount)	p.10-11		

For peer review only

Outcome data	15	*Cohort study* - Report numbers of outcome events or summary measures over time<br>*Case-control study* - Report numbers in each exposure category, or summary measures of exposure<br>*Cross-sectional study* - Report numbers of outcome events or summary measures	p.11		
Main results	16	(a) Give unadjusted estimates and, if applicable, confounder-adjusted estimates and their precision (e.g., 95% confidence interval). Make clear which confounders were adjusted for and why they were included<br>(b) Report category boundaries when continuous variables were categorized<br>(c) If relevant, consider translating estimates of relative risk into absolute risk for a meaningful time period	p.11-12 Table 4&5		
Other analyses	17	Report other analyses done—e.g., analyses of subgroups and interactions, and sensitivity analyses	N/A		
**Discussion**					
Key results	18	Summarise key results with reference to study objectives	p.12-13		
Limitations	19	Discuss limitations of the study, taking into account sources of potential bias or imprecision. Discuss both direction and magnitude of any potential bias	p.14	RECORD 19.1: Discuss the implications of using data that were not created or collected to answer the specific research question(s). Include discussion of misclassification bias, unmeasured confounding, missing data, and changing eligibility over	p.14

For peer review only

				time, as they pertain to the study being reported.	
Interpretation	20	Give a cautious overall interpretation of results considering objectives, limitations, multiplicity of analyses, results from similar studies, and other relevant evidence	p.12-14		
Generalisability	21	Discuss the generalisability (external validity) of the study results	p.12-14		
**Other Information**					
Funding	22	Give the source of funding and the role of the funders for the present study and, if applicable, for the original study on which the present article is based	p.15		
Accessibility of protocol, raw data, and programming code		p.16		RECORD 22.1: Authors should provide information on how to access any supplemental information such as the study protocol, raw data, or programming code.	p.16

*Reference: Benchimol EI, Smeeth L, Guttmann A, Harron K, Moher D, Petersen I, Sørensen HT, von Elm E, Langan SM, the RECORD Working Committee. The REporting of studies Conducted using Observational Routinely-collected health Data (RECORD) Statement. *PLoS Medicine* 2015; in press.

*Checklist is protected under Creative Commons Attribution (CC BY) license.

For peer review only

BMJ Open

Individual and contextual predictors of emergency department visits among community-living older adults: A register-based prospective cohort study

Journal:	*BMJ Open*
Manuscript ID	bmjopen-2021-055484.R1
Article Type:	Original research
Date Submitted by the Author:	01-Dec-2021
Complete List of Authors:	Naseer, Mahwish; Dalarna University, School of Health and Welfare; Karolinska Institutet, Aging Research Center, Department of Neurobiology, Care Sciences and Society<br>McKee, Kevin; Dalarna University, School of Health and Welfare<br>Ehrenberg, Anna ; Dalarna University, School of Health and Welfare<br>Schön, Pär; Karolinska Institutet, Aging Research Center, Department of Neurobiology, Care Sciences and Society<br>Dahlberg, Lena; Dalarna University, School of Health and Welfare; Karolinska Institutet, Aging Research Center, Department of Neurobiology, Care Sciences and Society
**Primary Subject Heading**:	Health services research
Secondary Subject Heading:	Emergency medicine, Geriatric medicine, Public health
Keywords:	PUBLIC HEALTH, International health services < HEALTH SERVICES ADMINISTRATION & MANAGEMENT, ACCIDENT & EMERGENCY MEDICINE

SCHOLARONE™
Manuscripts

I, the Submitting Author has the right to grant and does grant on behalf of all authors of the Work (as defined in the below author licence), an exclusive licence and/or a non-exclusive licence for contributions from authors who are: i) UK Crown employees; ii) where BMJ has agreed a CC-BY licence shall apply, and/or iii) in accordance with the terms applicable for US Federal Government officers or employees acting as part of their official duties; on a worldwide, perpetual, irrevocable, royalty-free basis to BMJ Publishing Group Ltd ("BMJ") its licensees and where the relevant Journal is co-owned by BMJ to the co-owners of the Journal, to publish the Work in this journal and any other BMJ products and to exploit all rights, as set out in our licence.

The Submitting Author accepts and understands that any supply made under these terms is made by BMJ to the Submitting Author unless you are acting as an employee on behalf of your employer or a postgraduate student of an affiliated institution which is paying any applicable article publishing charge ("APC") for Open Access articles. Where the Submitting Author wishes to make the Work available on an Open Access basis (and intends to pay the relevant APC), the terms of reuse of such Open Access shall be governed by a Creative Commons licence – details of these licences and which Creative Commons licence will apply to this Work are set out in our licence referred to above.

Other than as permitted in any relevant BMJ Author's Self Archiving Policies, I confirm this Work has not been accepted for publication elsewhere, is not being considered for publication elsewhere and does not duplicate material already published. I confirm all authors consent to publication of this Work and authorise the granting of this licence.

For peer review only

TITLE PAGE

Individual and contextual predictors of emergency department visits among community-living older adults: A register-based prospective cohort study

Running title: Predictors of emergency department visits

Mahwish Naseer[a,b] MSc, MPH, Kevin J. McKee[a] PhD, Anna Ehrenberg[a] PhD, Pär Schön[b] PhD, Lena Dahlberg[a,b] PhD

[a]School of Health and Welfare, Dalarna University, SE-79188 Falun, Sweden

[b]Aging Research Center, Karolinska Institutet & Stockholm University, Tomtebodavägen 18A, SE-17165 Solna, Sweden

Corresponding author

Mahwish Naseer

School of Health and Welfare, Dalarna University,

SE-79188 Falun, Sweden

mna@du.se

Word counts:

Abstract 255

Manuscript (excluding abstract, summary, and references) 3974

Number of tables 5

For peer review only - http://bmjopen.bmj.com/site/about/guidelines.xhtml

Abstract

Objectives: To examine the extent to which contextual factors explain emergency department (ED) visits and ED revisits, additional to that explained by individual factors.

Design: A register-based prospective cohort study.

Setting: Swedish region of Dalarna.

Participants: Participants were 16,543 community-living adults aged 80 or older who were residents of the Dalarna region of Sweden, excluding older adults who moved out of Dalarna or into residential care during the study period.

Outcome measures: Dependent variables were initial ED visit, and at least one ED revisit within 30 days of an initial ED visit.

Results: Approximately 36% of the participants visited the ED during the study period with 18.9% returning to the ED within 30 days. For both initial ED visits and ED revisits, the addition of contextual factors to models containing individual factors significantly improved model fit ($p<.001$; $p<.022$) and the amount of variance explained in the outcome. In the final models, initial ED visit was significantly associated with older age, number of chronic diseases, receipt of home help, number of primary care visits, proportion of 80+ in the population and shorter distance to the ED; while an ED revisit was significantly associated with greater use of social care, number of hospital admissions, and disposition (discharged; admitted to hospital) at initial ED visit.

Conclusion: Contextual factors explain variance in initial ED visit, additional to that explained by individual factors alone, which indicates inequitable access to ED care. These findings suggest considering local variations in contextual factors in order to improve health related outcomes among older adults.

For peer review only - http://bmjopen.bmj.com/site/about/guidelines.xhtml

Key words: health care use; acute care; home help use; Andersen model; older people

Strengths and limitations of this study

- Contextual factors were included in this study that sought to explain ED visits and revisits, this has rarely been done previously.
- This was a register-based cohort study with robust information on the health and social care use of the entire older adult population.
- Home help receipt was measured using both level and quality of home help.
- Administrative differences in how municipalities report data may impact the reliability of municipal level data.

For peer review only

1. Introduction

Emergency department (ED) visits are increasing worldwide, resulting in crowding and concern about the appropriateness of such visits.[1] A systematic review on ED visits found that older adults accounted for 12-21% of all ED visits.[2] In Sweden, adults aged 80 years or older constitute 5% of the total population yet account for 18% of all ED visits.[3] Older adults' complex health needs are associated with a relatively high use of health services but do not necessarily imply a requirement of ED care,[2] which is often poorly adapted to such needs and may carry a risk of negative outcomes.[4,5] While ED providers offer services/treatment for health problems and injuries that require immediate attention, some ED visits are partly explained by an unmet need for primary and social care for older adults[2,6]

The well-established Andersen model of health service use proposes the consideration of contextual factors along with individual factors for a better understanding of health care use.[7] Briefly, contextual factors include community characteristics and service provider-related factors that are measured at an aggregated level. Contextual factors, in the same way as individual factors, have been divided into three major components, that is, factors that predispose (e.g., proportion of 80+ in the total population), enable (e.g., supply of social and health care facilities), or approximate individual need for healthcare (e.g., disability rate).[7] In this study, we explored whether considering contextual factors in addition to individual factors can improve our understanding of ED visits among older adults.

Individual factors such as age, gender and health problems could potentially explain ED visits. There are mixed findings on the association between old age and ED visits, [8-10] possibly due to inconsistent methodology. There are higher odds of ED revisits among men.[8,11] Primary and social care play an important role in managing chronic diseases and in the proactive treatment of health problems that might require an ED visit.[2] However, research has shown that home help receipt and greater use of primary care are associated with ED revisits

For peer review only - http://bmjopen.bmj.com/site/about/guidelines.xhtml

and unplanned hospitalisations.[11, 12] While it is logical that greater need for care should be associated with greater use of services, service use does not necessarily ensure that care needs are met.[14] Low staff competence and poor continuity of care entails a risk of unmet needs[13] and may lead to avoidable ED visits. In Sweden, the proportion of registered nurses in municipal social care for older adults is lower than in neighbouring Norway (9% vs 31%),[15] while currently a person receiving home help meets an average of 16 different carers during a 14-day period.[3] Research investigating use of health care services among older adults has usually included a measure of home help receipt *per se*, rather than measuring its level (instrumental services, personal care or both) and quality .[11, 12, 16]

Contextual factors related to health care use include community characteristics such as the population age distribution, geographical location, and the organisation of health and social care.[7] In Sweden, health and social care is financed, managed and provided by 21 regions and 290 municipalities, with local variations in the management and delivery of care services. For example, without national guidelines on eligibility, municipalities decide on eligibility criteria for home help and residential care services.[17] This challenges the idea that all citizens in a universal welfare state should have equitable access to high quality care services driven by their need rather than, e.g., place of residence.[18, 19]

Health and social care systems internationally have undergone substantial changes. For example, Sweden has the lowest per capita hospital bed rate in Europe,[20] contributing to shorter lengths of hospital stay and an increased need of primary care, while there has been a 30 percent reduction in residential care since 2000.[17] A growing proportion of older adults are thus ageing at home and the availability of home help services is key to their living independently.[21] However, home help provision has not compensated for the decrease in residential care.[22] Instead, there has been an increase in informal care, particularly for older

For peer review only

adults with lower education, while those with higher education are more likely to buy private services.[18]

It is important to explore contextual factors potentially influencing ED use among older adults. Or and Penneau (2018) found that shorter distance to the ED and unavailability of primary care are associated with ED visits.[23] Studies on social care supply for older adults have shown that higher social care expenditures and greater availability of residential care are negatively associated with ED revisits.[21, 24] However, there are few studies on the association between home help coverage and ED visits.[21]

In summary, while both individual and contextual factors have the potential to explain ED care use in older adults, research has primarily focused on individual factors. Moreover, few studies on ED visits and revisits have considered the level and quality of home help receipt.

The aim of this study is to examine the extent to which contextual factors can improve the explanation of initial ED visits and ED revisits by community-living older adults, compared to that provided by individual factors alone.

2. Methods

2.1 Study design, setting and population

A prospective cohort design was used. The study population was community-living adults aged 80 or older who were residents of the Swedish region of Dalarna on 31st December 2014 (N=17,077). Excluding older adults who moved out of Dalarna (N=65) or into residential care during 2015 (N=469), the analytical sample was N =16,543.

There are fifteen municipalities in Dalarna whose population densities vary from 1.0 to 90.1 persons/km^2. Thus, great geographical variation is expected in health care and social care

For peer review only - http://bmjopen.bmj.com/site/about/guidelines.xhtml

access. There are four hospital EDs in Dalarna, all open 24-hours; one is regional, three are local, of which one is only for psychiatric emergencies.

2.2 Patient and public involvement

Patients or the public were not involved in the design or conduct of study.

2.3 Data sources

This study is based on data from three registers: two national (the Longitudinal Integration Database for Health Insurance and Labour Market (LISA) and the Social Services Register) and one regional (the health care database of Region Dalarna), all of which cover their respective entire populations. The registers were linked via encrypted personal numbers that are assigned to all individuals living in Sweden. Municipal-level data was accessed from the publicly available national database Kolada (https://www.kolada.se/), which is managed by the Council for the Promotion of Municipal Analyses.[25]

Ethical approval for the study was awarded by the Regional Ethical Review Board in Stockholm (reg.no. 2016/299-31).

2.4 Dependent variables (DVs)

There were two dependent variables: i) *initial ED visit* during 2015, and ii) at least one *ED revisit* within 30 days of an initial ED visit (both coded as no (0); yes (1)).[9, 11]. Information on visits at the four EDs was obtained from the regional database.

2.5 Independent variables (IVs)

Individual factors

Individual factors were categorised into two domains: a) demographic and health characteristics, and b) home help receipt and health care use.

For peer review only - http://bmjopen.bmj.com/site/about/guidelines.xhtml

Demographics and health: Information on *age* in 2014 based on date of birth, *gender*, and municipality of residence was obtained from LISA. Information on registered diagnosis (based on ICD-10 classification) at inpatient and outpatient visits during 2014 was obtained from the regional database and converted using a validated measure of chronic multi-morbidity in older adults into *number of chronic diseases*.[26]

Home help receipt and health care use: Information on *home help receipt* status at ED visit (or status in December 2014 for those who did not visit the ED during the study period) was obtained from the Social Services Register, which is updated monthly. Home help receipt status was measured as approvals of home help services. Home help receipt was categorised as no home help, only instrumental services (e.g., cleaning, shopping), only personal care (e.g., hygiene), and both instrumental services and personal care.

Information on number of *primary care visits*, *specialist care visits*, and *hospital admissions* 12 months prior to the initial ED visit (or during 2014 for those who did not visit the ED during the study period) and *disposition at ED visit* (discharged; admitted to hospital) was obtained from the regional database, the latter IV only analysed in relation to the ED revisit DV.

Contextual factors

Contextual factors covered municipality-level data from 2015 on: the *proportion of persons aged 80+ years* in the total population; annual *social care expenditures* per person aged 80+ years; *home help quality*; *median days in residential care* for adults aged 65+ resident in long-term care facilities; and *distance to the ED*. Distance to the ED which was estimated via Google maps and measured in kilometers as the shortest route from the centre of the municipality of residence to the nearest ED.[23] Since the ED in Säter is only for psychiatric care it was excluded in this estimation.

For peer review only - http://bmjopen.bmj.com/site/about/guidelines.xhtml

Two indicators were used of home help quality, based on data from an annual national survey distributed to adults aged 65+ years receiving home help and residential care.[27] Each indicator comprises the proportion of individuals within each municipality who provided only positive responses to questions on home help quality, with three questions in each of two domains: 1) *response, trust, and safety* (do home help staff usually respond well to you; do you trust the staff who come to your home; do you feel safe living in your home with the support of home help) and 2) *influence and adequate time* (do home help staff usually take account of your suggestions and wishes on how help should be provided; can you usually influence the time at which home help staff come to your place; do home help staff usually have adequate time to perform all their tasks at your place).

2.6 Data analysis

SPSS version 27 for Windows was used to conduct all analyses. Descriptive analyses were performed for all individual factors for the total sample and within municipalities, and for contextual factors at the municipality level. Odds ratios with 95% confidence intervals (CI) were calculated for the bivariate associations between individual and contextual factors (IVs) and ED visits and ED revisits (DVs).

Subsequently, all IVs with a significant bivariate association with the DVs were used to develop multivariable models of the DVs. First, since the data for ED visits and ED revisits were nested within municipalities, the relevance of nested models was tested by Generalized Linear Mixed Models.[28] The variance estimate at municipality level was 0.064 for initial ED visit and 0.019 for revisit, providing an intraclass coefficient (ICC) of 0.019 and 0.005. Thus, only 1.9% of the variance in initial visit and 0.57% in revisit was explained by municipality of residence, that is, considerably below the conventional level indicating a multilevel analysis is required.[29] Therefore, we proceeded with sequential logistic regression.

For peer review only - http://bmjopen.bmj.com/site/about/guidelines.xhtml

Model building proceeded in three steps. At the first step, individual demographic and health characteristics were entered in the models. At the second step, individual home help receipt and health care use were entered in the models and changes in model fit statistics examined. At the third step, contextual factors were entered in the models to determine if there was significant improvement in the models after entry of all individual factors. The deviance statistic (-2LL), Cox and Snell's R^2_{CS} and Nagelkerke R^2_N test were used for model fitting.[30] The deviance describes the unexplained variance in the model, so the smaller the value of deviance, the better the model fits the data. R^2_{CS} and R^2_N are approximations to the R^2 statistic for multiple linear regression which describes the variance in the DV explained by the model. R^2_{CS} and R^2_N are calculated differently and may provide divergent estimates, thus both were included.[30]

Variance inflation factor (VIF) was used to check potential multicollinearity among independent variables. VIF values were below 10 for all the independent variables and considered acceptable.[31]

3. Results

3.1 Descriptive analyses

The mean age of the participants was 85.2 years and 58.5% were women (Table 1). The mean number of chronic diseases was 2.1 (range 1.8-2.4 across municipalities). Seventy-four percent lived at home with no home help, while 5.1% received instrumental services only, 3.9% personal care only, and 16.3% received instrumental services and personal care (range for home help receipt 19.1%-37.1%).

The mean number of primary care visits was 9.0 (range 7.5-10.4), while the mean number of specialist care visits was 2.7 (range 1.7-4.1) (Table 2). The mean number of hospital admissions was 0.3 (range 0.3-0.4). In the sample, 36.4% had at least one ED visit, of whom

For peer review only - http://bmjopen.bmj.com/site/about/guidelines.xhtml

18.9% had an ED revisit within 30 days. The mean number of ED visits was 0.7 (range 0.4-0.9).

Contextual factors are presented in Table 3. The proportion of adults aged 80+ years varied across municipalities from 5.3% to 9.0%, while social care costs per person varied from Swedish Korona (SEK) 207,741 to SEK 272,317 during 2015 (approx. $ 25,000-32,000). Positive home help quality varied between 35.0 % and 56.0% for response, trust, and safety and between 34.0% and 60.0% for influence and adequate time. Median days in residential care varied between 407 and 867, while distance to the nearest ED varied from 1.8 km to 78.8 km.

3.2 Initial ED visit

In bivariate analyses, individual factors significantly positively associated with initial ED visit were: age, number of chronic diseases, home help receipt, number of primary care visits, specialist care visits, and hospital admissions (Table 4). Contextual factors significantly negatively associated with ED visits were: median days in residential care, proportion of adults 80+ years in the population and greater distance to the ED.

In model 1 with the demographic and health characteristics age and number of chronic conditions included, deviance=20624.7, R^2_{CS}=.062 and R^2_{N}=.085. Compared to a constant-only model, model 1 is a significant improvement ($\chi^2(2)$=1065.2, p<.001). In model 2 with the addition of variables measuring home help and health care use, deviance=19883.9, R^2_{CS}=.103 and R^2_{N}=.142. Compared to model 1, model 2 is a significant improvement (model $\chi^2(8)$=1806.1, p<.001; step $\chi^2(6)$=740.9, p<.001). In model 3 with the addition of variables measuring contextual factors, deviance=19701.7, R^2_{CS}=.113 and R^2_{N}=.155. Compared to model 2, model 3 is a significant improvement (model $\chi^2(11)$=1988.3, p<.001; step $\chi^2(3)$=182.2, p<.001. Thus, the multivariable models indicated a better fit at each step.

For peer review only - http://bmjopen.bmj.com/site/about/guidelines.xhtml

In the final multivariable model (model 3), individual level factors with significant higher odds ratios for ED visits were: age, number of chronic diseases, home help receipt, and number of primary care visits. Contextual factors significantly associated with ED visit were: higher proportion of adults 80+ years in the total population and shorter distance to the ED.

3.3 ED revisit

In bivariate analyses, number of chronic diseases, receipt of both instrumental and personal care from home help services, number of primary care visits and hospital admissions prior to initial ED visit were significantly positively associated with ED revisit, while being admitted to hospital at initial ED visit was significantly negatively associated with an ED revisit. (Table 5). Of the contextual factors, distance to the ED was significantly negatively associated with an ED revisit.

In model 1 with the health characteristic number of chronic conditions included, deviance=5718.9, R^2_{CS}=.004 and R^2_N=.006. Compared to a constant-only model, model 1 is a significant improvement ($\chi^2(1)$=22.4, p<.001). In model 2 with the addition of variables measuring home help and health care use, deviance=5650.0, R^2_{CS}=.015 and R^2_N=.025. Compared to model 1, model 2 is a significant improvement (model $\chi^2(7)$=91.3, p<.001; step $\chi^2(6)$=68.9, p<.001). In model 3 with the addition of the contextual factor distance to the nearest ED, deviance=5644.8, R^2_{CS}=.016 and R^2_N=.026. Compared to model 2, model 3 is a significant improvement (model $\chi^2(8)$=96, p<.001; step $\chi^2(1)$=5.26, p<.022). Thus, the multivariable models indicated a better fit at each step.

In the final multivariable model (model 3), receipt of both instrumental and personal care from home help services, number of hospital admissions, and disposition at initial ED visit were significantly associated with ED revisits. Although the addition of distance to nearest ED in model 3 resulted in a significant improvement in model fit compared to model 2, as the

For peer review only - http://bmjopen.bmj.com/site/about/guidelines.xhtml

CIs of the odds ratio for the variable contains 1 we conclude that the association between distance to ED and ED revisit was not significant in the model.

4. Discussion

4.1 Main findings

Approximately 36% of the participants visited the ED, of whom 18.9% had an ED revisit within 30 days. Research has mainly focused on individual predictors of ED care. Our study indicates that ED care can be further explained by contextual factors. Contextual factors associated with higher odds of initial ED visit indicate inequitable access to ED care. Receipt of both instrumental services and personal care is an important predictor of ED visits and revisits.

4.2 Comparison with other studies

Our findings on the associations between age, number of chronic diseases, and initial ED visits are in line with Andersen's model, which describes equitable access to ED care explained by demographic factors (e.g., age) and need (e.g., chronic diseases).[7] Health decline with age and multimorbidity relates to the increasing need for health care. Grunier et al. (2011) argued that social and primary care can play a role in the management of need and can contribute positively to reducing ED visits. Our study shows that the addition of home help and health care variables increased the variance explained in DVs by demographics and health characteristics and improved the overall model fit. Positive associations between initial ED visit and both home help receipt and primary care visits suggest that the provided services do not meet the recipient's care needs [2], that is, indicate a poorly functioning health and social care system. However, extensive home help provision is also an indication of poor health/functional status with an increased risk for ED visits.

For peer review only - http://bmjopen.bmj.com/site/about/guidelines.xhtml

The needs of older adults are often complex, and inadequate care co-ordination between hospitals, primary and municipal social care for older adults increases the risk of negative health outcomes. In Sweden, as in other high-income countries, the fragmentation of health and social care systems has become more visible during the COVID-19 pandemic, and there is a need to establish channels that ensure continuous and rapid coordination of services from different care providers and levels of organisations.[32, 33] Research has shown that integrated care, i.e., co-ordination between different care providers, can reduce ED visits, hospital admissions, and the length of hospital stay.[34, 35]

The ED visit models explain more variance in the outcome than the revisit model and more IVs are significant in the model, including contextual factors. This may be partly explained by the revisit models having less variation in its DV, and perhaps also due to ED revisits more than initial ED visit being related to extraneous variables that were not available in the registers accessed and thus not included in the models. The odds of revisits were higher in those who were not admitted to inpatient hospital care at initial visit and among those with greater use of home help services and higher number of hospital admissions. With shorter lengths of hospital stay,[20] home help providers have to care for older adults with more complex care needs.[3] A recent report showed that patients who were sent home after initial ED visit had a declining health status at ED revisit within 30 days, i.e., that their needs for aftercare were not met.[36]

Our results show that contextual factors (living in municipalities with higher proportions of adults aged 80+ in the population and a shorter distance to the ED) increased the odds of an initial ED visit at the individual level. Critical events that require ED care are common in the 80+ group due to multiple health problems, complex needs, and rapid changes in health status, which put pressure on the local health care system and this may explain the association between a higher proportion of 80+ in the population and ED visits. The association between

For peer review only - http://bmjopen.bmj.com/site/about/guidelines.xhtml

distance to the ED and ED visits raises concern on the appropriateness of such visits. Access to ED care should be driven by need rather than place of residence, and the variance in initial ED visit explained by contextual factors challenges the idea of universalism and equitable access to health care. This suggests that considering the healthcare needs of the local population and geographical variation in healthcare planning may improve the accessibility of primary and social care for older adults.[23]

4.3 Implications

Those individuals who receive both instrumental services and personal care constitute a vulnerable group that should be considered in interventions for reducing ED visits, discharge planning, and aftercare. Considering local variations in healthcare needs and access to health and social care is important for improving health-related outcomes among older adults.

4.4 Strengths and limitations

A strength of this study was that it was based on register data. Registers provide high quality information on health and social care use covering the entire population. This study also included contextual factors in seeking to explain ED visits, which have been rarely considered previously, as well as levels and quality of home help receipt as factors in ED visits rather than simply use/non-use.

Home help receipt was categorised as an individual factor, while home help quality was categorised as a contextual variable as the individual-level data was only available for analysis as aggregated data. Thus, some variables that might explain ED visits or revisits can be optionally conceptualised and operationalised at individual or contextual levels. An association between variables can differ in strength and direction when analysed at individual versus group level, so it is important that the use of aggregated data as a contextual factor can be defended. The reliability of the data on home help quality will have been influenced by a

For peer review only - http://bmjopen.bmj.com/site/about/guidelines.xhtml

For peer review only

survey response rate that varied between 48-66% by municipality and only 46% of the data provided by care recipients with the rest provided by relatives or care providers.

The measure of the number of chronic diseases used in this study included healthcare use in 2014 only. Patients who were admitted to the hospital compared to those discharged after the initial ED visit did not have an equal chance to be included within the 30 days criterion for an ED revisit. This problem is minor, though, since the median length of stay in hospital was 4 days. Administrative differences in how municipalities report data to the Kolada database may impact data reliability. Distance to the nearest ED was estimated from the city centre rather than place of residence. Finally, the amount of variance our models explained in their respective outcomes was not substantial, this was particularly the case for ED revisits. This is perhaps not surprising given the many factors that might potentially influence ED use and ED revisits, and indicates that future research should seek to incorporate a broader range of variables than were available for the present study to advance the understanding of ED visits and revisits.

5. Conclusion

The aim of this study was to consider whether contextual factors improve our understanding of ED visits. Results suggest that contextual factors explain additional significant variance in initial ED visit compared to that explained by individual factors alone, which indicates inequitable access to ED care.

In individual factors, age and health characteristics explain variance in initial visit while receipt of home help and health care use explained variance both in initial visit and revisit. ED revisit was also explained by discharge from ED and can be interpreted as a result of inadequate care and discharge planning at the initial visit. Greater use of home help and primary care does not ensure that needs are met, and further research on the impact of unmet

For peer review only - http://bmjopen.bmj.com/site/about/guidelines.xhtml

care needs in older adults on ED visits can clarify how the use of different health and social care services are associated. Local variations in home help receipt and utilisation of ED care challenge the notion of a universal welfare state, and require consideration in order to improve health related outcomes among older adults.

Funding: The study was supported by Swedish Research Council for Health, Working Life and Welfare (grant no. 2015-00440) and Region Dalarna. The funding organisation had no role in the development of study design, analysis, and/or in manuscript writing.

Conflict of Interest: The authors declare that they have no conflict of interest.

Author Contribution: Planning and study design (MN, KJM, AE, PS, LD), conducted data analysis (MN), advice regarding data analysis (KJM) interpreting analyses and results (MN, KJM, AE, PS, LD), drafting of manuscript (MN), revising and finalising the manuscript (MN, KJM, AE, PS, LD), final approval of the manuscript (MN, KJM, AE, PS, LD).

Patient consent: Not applicable

Data availability statement: Data may be obtained from a third party and are not publicly available.

References

1. Boyle A, Beniuk K, Higginson I, Atkinson P. Emergency department crowding: time for interventions and policy evaluations. Emerg Med Int. 2012; 2012:838610.

2. Gruneir A, Silver MJ, Rochon PA. Emergency department use by older adults: A literature review on trends, appropriateness, and consequences of unmet health care needs. Med Care Res Rev 2011; 68(2): 131-155.

For peer review only - http://bmjopen.bmj.com/site/about/guidelines.xhtml

3. National Board of Health and Welfare. Vård och omsorg om äldre lägesrapport 2020 [The national report on social care for older adults 2020]. Stockholm: National Board of Health and Welfare.

4. Covinsky KE, Pierluissi E, Johnston CB. Hospitalization-associated disability: "She was probably able to ambulate, but I'm not sure". JAMA 2011; 306(16): 1782–1793.

5. SBU. Omhändertagande av äldre som inkommer akut till sjukhus-med fokus på sköra äldre. En systematisk litteratur översikt [Care of older people coming to hospital emergency: a focus on frail older people. A systematic literature review] 2013. Stockholm: Swedish Agency for Health Technology Assessment.

6. Hass Z, DePalma G, Craig BA, Xu H, Sands LP. Unmet need for help with activities of daily living disabilities and emergency department admissions among older medicare recipients. Gerontologist 2017; 57(2): 206-210.

7. Andersen RM. National Health Surveys and the Behavioral Model of Health Services Use. Med Care 2008; 46(7): 647-653.

8.de Gelder J, Lucke JA, de Groot B et al. Predictors and outcomes of revisits in older adults discharged from the emergency department. J Am Geriatr Soc 2018; 66(4): 735-741.

9. Lowthian J, Straney LD, Brand CA et al. Unplanned early return to the emergency department by older patients: the Safe Elderly Emergency Department Discharge (SEED) project. Age Ageing 2016; 45(2): 255-261.

10.Lynch B, Fitzgerald AP, Corcoran P, Buckley C, Healy O, Browne J. Drivers of potentially avoidable emergency admissions in Ireland: an ecological analysis. BMJ Qual Saf 2019; 28(6): 438-448.

For peer review only

For peer review only - http://bmjopen.bmj.com/site/about/guidelines.xhtml

11. Naseer M, Agerholm J, Fastbom J, Schön P, Ehrenberg A, Dahlberg L. Factors associated with emergency department revisits among older adults in two Swedish regions: A prospective cohort study. Arch Gerontol Geriatr 2020; 86: 103960.

12. Dahlberg L, Agahi N, Schön P, Lennartsson C. Planned and unplanned hospital admissions and their relationship with social factors: findings from a national, prospective study of people aged 76 years or older. Health Ser Res 2018; 53(6): 4248-4267.

13. Bravell ME, Bennich M, Walfridsson C. 'In August, I counted 24 different names': Swedish older adults' experiences of home care. J Appl Gerontol 2021; 40(9): 1020-1028.

14. Cunningham A, Mautner D, Ku B, Scott K, LaNoue M. Frequent emergency department visitors are frequent primary care visitors and report unmet primary care needs. J Eval Clin Pract 2017; 23(3): 567-573.

15. SOU. Internationella erfarenheter av Covid-19 i äldreboende [International experiences of COVID-19 in residential care] 2020. Reports SOU Stockholm 2020: 80.

16. Tanderup A, Ryg J, Rosholm JU, Lassen AT. Association between the level of municipality healthcare services and outcome among acutely older patients in the emergency department: a Danish population-based cohort study. BMJ Open 2019; 9(4): e026881.

17. Schön, P., & Heap, J. European Social Policy Network (ESPN) Thematic report on challenges in long-term care Sweden 2018. Available at: https://ec.europa.eu/social/main.jsp?pager.offset=30&catId=792&langId=en&moreDocuments=yes. Accessed October 12, 2020.

18. Szebehely M, Trydegård GB. Home care for older people in Sweden: a universal model in transition. Health Soc Care Community 2012; 20(3): 300-309.

For peer review only

19. Trydegård GB, Thorslund M. One uniform welfare state or a multitude of welfare municipalities? The evolution of local variation in Swedish elder care. Soc Policy Adm 2010; 44(4); 495-511.

20. OECD. Health at a glance: Europe 2020. State of Health in the EU Cycle. OECD Publishing.

21. Spiers G, Matthews FE, Moffatt S, et al. Impact of social care supply on healthcare utilisation by older adults: a systematic review and meta-analysis. Age Ageing 2018; 48(1); 57-66.

22. Ulmanen P, Szebehely M. From the state to the family or to the market? Consequences of reduced residential eldercare in Sweden. Int J Soc Welfare 2015; 24: 81-92.

23. Or Z, Penneau A. A Multilevel Analysis of the determinants of emergency care visits by the elderly in France. Health Policy 2018; 122(8): 908-914.

24. Fernandez JL, Forder J. Consequences of local variations in social care on the performance of the acute health care sector. Appl Econ 2008; 40(12): 1503-1518.

[dataset] 25. Council for the Promotion of Municipal Analyses. Data from: Kolada; Den öppna och kostnadsfria databasen för kommuner och regioner [The free database for municipalities and regions] 2015. Kolada, December 1st, 2021. https://www.kolada.se/

26. Calderón-Larrañaga A, Vetrano DL, Onder G et al. Assessing and measuring chronic multimorbidity in the older population: A proposal for its operationalization. J Gerontol A Biol Sci Med Sci 2017; 72(10): 1417-1423.

27. National Board of Health and Welfare. Så tycker de äldre om äldreomsorgen 2015: En rikstäckande undersökning i hemtjänst och äldreboenden [What older people thinks on

For peer review only - http://bmjopen.bmj.com/site/about/guidelines.xhtml

eldercare 2015: A national survey on home help and institutional care]. Stockholm: National Board of Health and Welfare.

28. Sommet N, Morseli D. Keep calm and learn multilevel logistic modelling: A simplified three-step procedures using Stata, R, Mplus, and SPSS. International Review Social Psychology 2017; 30 1(1): 203-218.

29. Kreft IGG, de Leeuw J. Introducing multilevel modeling, SAGE Publications, 1998.

30. Field A. Discovering statistics using IBM SPSS Statistics. 4th Ed. London: SAGE Publications, 2013.

31.Myers R. Classical and modern regression with applications. 1990. In: Field A. Discovering statistics using IBM SPSS Statistics. 4th Ed. London: SAGE Publications, 2016.

32. SOU. The elderly care in the pandemic 2020. Corona commission: Statens Offentliga Utredningar, Stockholm 2020 80. Available at: https://coronakommissionen.com/. Accessed January 30, 2021.

33. Estabrooks CA, Straus S, Flood, CM, et al. Restoring trust: COVID-19 and the future of long-term care. Royal Society of Canada. 2020. Available at: https://rsc-src.ca/en/themes/long-term-care. Accessed May 21st, 2021.

34. Liljas AEM, Brattström F, Burström B, Schön P, Agerholm J. Impact of integrated care on patient-related outcomes among older people-A systematic review. Int J Integr Care 2019; 19(3): 6, 1-6.

35. Dohney M, Agerholm J, Orsini N, Schön P, Burström B. Impact of integrated care on trends in the rate of emergency department visits among older persons in Stockholm county: an interrupted time series analysis. BMJ Open 2020; 10: e0361182.

For peer review only

For peer review only - http://bmjopen.bmj.com/site/about/guidelines.xhtml

36. Stockholm Gerontology Research Center. Äldres resa till, genom och ut från sjukhuset i Stockholms län: En lägesbeskrivning inför lagen om samverkan vid utskrivning från sluten hälso-och sjukvård [Older people's way through hospital care in Region Stockholm: A description of the situation before the implementation of the Act on coordinated discharge from hospital care] 2018. Reports Stockholm Gerontology Research Center 2018 1.

For peer review only

For peer review only - http://bmjopen.bmj.com/site/about/guidelines.xhtml

Table 1

Demographic, health and home help use characteristics for total study participants (N=16,543) and stratified by municipality

Municipality	n	Demographics		Health	Home help			
		Age	Female gender	No. of chronic diseases	No	Instrumental services	Personal care	Instrumental & personal care
		Mean (SD)		Mean (SD)	%	%	%	%
Avesta	1474	85.1 (4.1)	57.1	2.4 (2.0)	80.5	3.1	2.1	14.2
Borlänge	2599	85.1 (4.2)	57.5	2.0 (1.8)	72.0	1.3	12.	14.2
Falun	2925	85.2 (4.2)	58.5	2.2 (1.8)	62.9	14.0	1.1	22.0
Gagnef	539	85.1 (4.0)	54.2	2.1 (1.7)	79.8	2.0	4.0	14.1
Hedemora	932	85.1 (4.2)	59.5	2.2 (1.9)	78.8	2.3	2.0	16.8
Leksand	945	85.4 (4.2)	59.0	1.8 (1.7)	78.7	2.7	2.7	15.9
Ludvika	1732	85.5 (4.3)	60.9	2.1 (1.8)	75.6	6.6	2.1	15.6
Malung-Sälen	633	84.8 (4.1)	59.1	2.2 (1.9)	77.3	3.4	2.3	16.9
Mora	1215	85.4 (4.4)	59.6	2.0 (1.9)	78.2	2.2	2.8	16.7
Orsa	477	85.1 (4.1)	58.5	2.3 (2.0)	78.8	2.5	3.1	15.5
Rättvik	905	85.3 (4.1)	60.0	1.9 (1.8)	80.8	4.2	1.5	13.5
Smedjebacken	617	84.8 (4.0)	55.9	2.1 (1.8)	78.1	6.0	4.5	11.3
Säter	591	85.2 (4.2)	57.9	2.1 (1.8)	76.3	4.0	3.0	16.6
Vansbro	440	84.9 (4.0)	58.6	2.1 (1.9)	80.9	0.9	4.7	13.4
Älvdalen	519	85.0 (4.0)	59.0	2.2 (1.9)	75.7	4.2	3.6	16.4
Total	16,543	85.2 (4.2)	58.5	2.1 (1.8)	74.6	5.1	3.9	16.3

For peer review only

Table 2:

Health care use for participants (N=16,543) and stratified by municipality.

Municipality	n	No. of primary care visits	No. of specialist care visits	No. of hospital admissions	ED visits (Yes)	No. of ED visits	¤ED revisit
		Mean (SD)	Mean (SD)	Mean (SD)	%	Mean (SD)	%
Avesta	1474	7.5 (8.0)	3.0 (8.1)	0.4 (0.9)	38.9	0.8 (1.5)	23.5
Borlänge	2599	8.6 (9.3)	2.6 (8.5)	0.3 (0.8)	36.0	0.6 (1.1)	20.0
Falun	2925	10.4 (10.8)	2.9 (9.3)	0.3 (0.8)	41.8	0.8 (1.6)	19.1
Gagnef	539	8.4 (10.3)	2.2 (6.6)	0.3 (0.8)	34.0	0.6 (1.2)	21.3
Hedemora	932	8.1 (8.5)	2.5 (7.1)	0.3 (0.8)	36.8	0.7 (1.4)	22.3
Leksand	945	10.2 (10.9)	1.9 (3.7)	0.3 (0.8)	36.2	0.7 (1.3)	16.2
Ludvika	1732	8.8 (9.8)	3.0 (9.0)	0.3 (0.8)	26.2	0.4 (0.9)	15.6
Malung-Sälen	633	9.2 (9.0)	2.0 (7.4)	0.3 (0.7)	28.1	0.4 (0.9)	13.0
Mora	1215	9.2 (8.6)	3.3 (9.4)	0.4 (0.9)	44.7	0.9 (1.6)	17.8
Orsa	477	8.4 (8.7)	4.1 (16.8)	0.4 (0.8)	44.9	0.8 (1.4)	17.8
Rättvik	905	8.4 (9.0)	2.3 (6.4)	0.3 (0.8)	37.2	0.7 (1.2)	21.1
Älvdalen	519	8.5 (9.2)	2.6 (12.2)	0.3 (0.7)	38.2	0.6 (1.1)	17.6
Smedjebacken	617	8.9 (8.3)	2.2 (5.5)	0.3 (0.8)	26.1	0.4 (0.9)	15.0
Säter	591	9.2 (9.8)	1.7 (3.8)	0.3 (0.7)	33.3	0.5 (1.1)	13.9
Vansbro	440	9.8 (9.6)	2.2 (7.0)	0.3 (0.8)	31.4	0.5 (1.1)	18.4
Älvdalen	519	8.5 (9.2)	2.6 (12.2)	0.3 (0.7)	38.2	0.6 (1.1)	17.6
Total	16,543	9.0 (9.6)	2.7 (8.5)	0.3 (0.8)	36.4	0.7 (1.3)	18.9

Note: Abbreviations: ED, emergency department; SD, standard deviation

¤ Sample for ED revisit includes only those who visited ED (n = 5930).

For peer review only

Table 3

Contextual factors at municipality level

Municipality	Proportion of 80+ in the population	Annual social care cost per person 80+	Positive home help quality		Median number of days in residential care	Distance to the nearest ED
			Response, trust, & safety	Influence & adequate time		
	%	SEK	%	%		km
Avesta	6.7	207,741	41.0	44.0	578	2.3
Borlänge	5.3	229,601	37.0	41.0	585	21.3
Falun	5.4	244,487	48.0	60.0	573	1.8
Gagnef	5.7	255,825	44.0	57.0	610	40.2
Hedemora	6.3	218,694	40.0	53.0	680	20.9
Leksand	6.7	235,201	50.0	51.0	561	49.3
Ludvika	7.0	224,179	42.0	46.0	715	64.4
Malung-Sälen	7.0	223,591	51.0	55.0	867	73.0
Mora	6.4	250,077	40.0	46.0	407	2.00
Orsa	7.3	221,582	37.0	34.0	740	12.1
Rättvik	9.0	209,722	45.0	49.0	597	35.6
Smedjebacken	6.1	215,149	39.0	40.0	647	60.8
Säter	5.8	229,107	56.0	53.0	506	43.2
Vansbro	7.0	219,152	35.0	39.0	828	78.8
Älvdalen	7.5	272,317	52.0	49.0	711	41.7

For peer review only

Table 4

Models for binary logistic regression of factors associated with initial ED visit (N = 16,543)

	ED visit			
	Bivariate OR (95% CI)	Model 1 OR (95% CI)	Model 2 OR (95% CI)	Model 3 OR (95% CI)
Individual factors				
**Demographics & health**				
Age	1.08 (1.07-1.09)*	1.09 (1.08-1.09)*	1.06 (1.05-1.06)*	1.06 (1.05-1.07)*
Gender				
Female	Ref.			
Male	1.04 (0.97-1.11)			
No. of chronic diseases	1.22 (1.20-1.25)*	1.24 (1.21-1.26)*	1.12 (1.10-1.15)*	1.12 (1.10-1.15)*
**Home help & health care use**				
Home help				
No	Ref.		Ref.	Ref.
Instrumental services	1.74 (1.51-2.01)*		1.31 (1.13-1.52)*	1.26 (1.08-1.46)*
Personal care	2.00 (1.71-2.34)*		1.49 (1.26-1.76)*	1.55 (1.31-1.83)*
Instrumental & personal care	3.38 (3.10-3.68)*		2.49 (2.27-2.74)*	2.48 (2.26-2.73)*
No. of primary care visits	1.05 (1.04-1.05)*		1.03 (1.03-1.04)*	1.03 (1.03-1.04)*
No. of specialist care visits	1.02 (1.01-1.02)*		1.00 (1.00-1.01)	1.00 (1.00-1.01)
No. of hospital admissions	1.37 (1.32-1.42)*		1.01 (0.96-1.05)	1.00 (0.96-1.05)
Contextual factors				
Annual social care cost per person 80+	1.00 (1.00-1.00)			
Response, trust, & safety	1.00 (0.99-1.00)			
Influence & adequate time	1.00 (1.00-1.01)			
Median days in residential care	0.99 (0.99-0.99)*			1.00 (0.99-1.00)
Proportion of 80+ in the population	0.94 (0.91-0.98)*			1.09 (1.05-1.13)*
Distance to the nearest ED	0.99 (0.99-0.99)*			0.99 (0.98-0.99)*
Deviance (-2LL)		20624.749	19883.865	19701.645
Cox & Snell R^2_{CS}		0.062	0.103	0.113
Nagelkerke R^2_N		0.085	0.142	0.155

Note: The negative bivariate association for proportion of 80+ in the population with initial ED visit becomes a positive association and increases in magnitude in the multivariable model, indicating negative suppression. Through trial variable removal from the model,[30] it was determined that distance to the ED and median days in residential care both acted as suppressor variables for proportion 80+ in the population.
Abbreviations: ED, emergency department; OR, odds ratio; CI, confidence interval
*Significant on values estimated from 95% CI

For peer review only

Table 5

Models for logistic regression of factors associated with ED revisit within 30 days after initial ED visit (n =5930)

	**ED revisit**			
	Bivariate OR (95% CI)	Model 1 OR (95% CI)	Model 2 OR (95% CI)	Model 3 OR (95% CI)
Individual factors				
**Demographics & health**				
Age	0.99 (0.98-1.01)			
Gender				
Female	Ref.			
Male	1.12 (0.98-1.27)			
No. of chronic diseases	1.07 (1.04-1.10)*	1.07 (1.04-1.10)*	1.02 (0.98-1.06)	1.02 (0.98-1.06)
**Home help & health care use**				
Home help				
No	Ref.		Ref.	Ref.
Instrumental services	0.99 (0.75-1.32)		0.99 (0.74-1.32)	0.97 (0.73-1.29)
Personal care	1.13 (0.84-1.53)		1.13 (0.84-1.53)	1.15 (0.85-1.55)
Instrumental & personal care	1.33 (1.15-1.55)*		1.31 (1.13-1.53)*	1.30 (1.12-1.51)*
No. of primary care visits	1.01 (1.01-1.02)*		1.00 (1.00-1.01)	1.00 (1.00-1.01)
No. of specialist care visits	1.00 (0.99-1.00)			
No. of hospital admissions	1.19 (1.13-1.26)*		1.15 (1.08-1.23)*	1.15 (1.08-1.23)*
Disposition at index ED visit				
Discharged	Ref.		Ref.	Ref.
Hospital admission	0.73 (0.65-0.83)*		0.69 (0.61-0.79)*	0.71 (0.62-0.81)*
Contextual factors				
Annual social care cost per person 80+	1.00 (1.00-1.00)			
Response, trust, & safety	0.98 (0.97-1.00)			
Influence & adequate time	0.99 (0.99-1.00)			
Median days in residential care	1.00 (0.99-1.00)			
Propotion of 80+ in the population	0.99 (0.92-1.06)			
Distance to the nearest ED	0.99 (0.99-0.99)*			0.99 (0.99-1.00)
Deviance (-2LL)		5718.922	5650.039	5644.775
Cox & Snell R^2_{CS}		0.004	0.015	0.016
NagelkerkeR^2_N		0.006	0.025	0.026

Note: Abbreviations: ED, emergency department; OR, odds ratio; CI, confidence interval

*Significant on values estimated from 95% CI

For peer review only - http://bmjopen.bmj.com/site/about/guidelines.xhtml

The RECORD statement – checklist of items, extended from the STROBE statement, that should be reported in observational studies using routinely collected health data.

	Item No.	STROBE items	Location in manuscript where items are reported	RECORD items	Location in manuscript where items are reported
**Title and abstract**					
	1	(a) Indicate the study's design with a commonly used term in the title or the abstract (b) Provide in the abstract an informative and balanced summary of what was done and what was found	a) Title and abstract (sub-heading: design)<br>b) Abstract (p.2)	RECORD 1.1: The type of data used should be specified in the title or abstract. When possible, the name of the databases used should be included.<br><br>RECORD 1.2: If applicable, the geographic region and timeframe within which the study took place should be reported in the title or abstract.<br><br>RECORD 1.3: If linkage between databases was conducted for the study, this should be clearly stated in the title or abstract.	Title and abstract (register)<br><br>Abstract<br><br>Linkage between databases was conducted for independent variables, therefore mentioned in the main text under subheading data sources (p. 7)
**Introduction**					
Background rationale	2	Explain the scientific background and rationale for the investigation being reported	p.4-6		
Objectives	3	State specific objectives, including any prespecified hypotheses	p.6		
**Methods**					

For peer review only

Study Design	4	Present key elements of study design early in the paper	p.6		
Setting	5	Describe the setting, locations, and relevant dates, including periods of recruitment, exposure, follow-up, and data collection	p.6		
Participants	6	*(a) Cohort study* - Give the eligibility criteria, and the sources and methods of selection of participants. Describe methods of follow-up<br>*Case-control study* - Give the eligibility criteria, and the sources and methods of case ascertainment and control selection. Give the rationale for the choice of cases and controls<br>*Cross-sectional study* - Give the eligibility criteria, and the sources and methods of selection of participants<br><br>*(b) Cohort study* - For matched studies, give matching criteria and number of exposed and unexposed<br>*Case-control study* - For matched studies, give matching criteria and the number of controls per case	p.6-7	RECORD 6.1: The methods of study population selection (such as codes or algorithms used to identify subjects) should be listed in detail. If this is not possible, an explanation should be provided.<br><br>RECORD 6.2: Any validation studies of the codes or algorithms used to select the population should be referenced. If validation was conducted for this study and not published elsewhere, detailed methods and results should be provided.<br><br>RECORD 6.3: If the study involved linkage of databases, consider use of a flow diagram or other graphical display to demonstrate the data linkage process, including the number of individuals with linked data at each stage.	N/A (register-based study covering entire population)<br><br>N/A<br><br>Register-based study covering entire population
Variables	7	Clearly define all outcomes, exposures, predictors, potential confounders, and effect modifiers. Give diagnostic criteria, if applicable.	p.7-8	RECORD 7.1: A complete list of codes and algorithms used to classify exposures, outcomes, confounders, and effect modifiers should be provided. If these cannot be reported, an explanation should be provided.	p.7-8
Data sources/ measurement	8	For each variable of interest, give sources of data and details	p.6-8		

For peer review only

		of methods of assessment (measurement). Describe comparability of assessment methods if there is more than one group			
Bias	9	Describe any efforts to address potential sources of bias	N/A		
Study size	10	Explain how the study size was arrived at	N/A (entire population)		
Quantitative variables	11	Explain how quantitative variables were handled in the analyses. If applicable, describe which groupings were chosen, and why	p.7-8		
Statistical methods	12	(a) Describe all statistical methods, including those used to control for confounding<br>(b) Describe any methods used to examine subgroups and interactions<br>(c) Explain how missing data were addressed<br>(d) *Cohort study* - If applicable, explain how loss to follow-up was addressed<br>*Case-control study* - If applicable, explain how matching of cases and controls was addressed<br>*Cross-sectional study* - If applicable, describe analytical methods taking account of sampling strategy<br>(e) Describe any sensitivity analyses	p.8-9		
Data access and cleaning methods		..		RECORD 12.1: Authors should describe the extent to which the investigators had access to the database	p.6-7

For peer review only

				population used to create the study population.<br><br>RECORD 12.2: Authors should provide information on the data cleaning methods used in the study.	
Linkage		..		RECORD 12.3: State whether the study included person-level, institutional-level, or other data linkage across two or more databases. The methods of linkage and methods of linkage quality evaluation should be provided.	p.6-7
**Results**					
Participants	13	(a) Report the numbers of individuals at each stage of the study (*e.g.*, numbers potentially eligible, examined for eligibility, confirmed eligible, included in the study, completing follow-up, and analysed)<br>(b) Give reasons for non-participation at each stage.<br>(c) Consider use of a flow diagram	p.6	RECORD 13.1: Describe in detail the selection of the persons included in the study (*i.e.,* study population selection) including filtering based on data quality, data availability and linkage. The selection of included persons can be described in the text and/or by means of the study flow diagram.	p.6
Descriptive data	14	(a) Give characteristics of study participants (*e.g.*, demographic, clinical, social) and information on exposures and potential confounders<br>(b) Indicate the number of participants with missing data for each variable of interest<br>(c) *Cohort study* - summarise follow-up time (*e.g.*, average and total amount)	p.10-11		

Outcome data	15	*Cohort study* - Report numbers of outcome events or summary measures over time<br>*Case-control study* - Report numbers in each exposure category, or summary measures of exposure<br>*Cross-sectional study* - Report numbers of outcome events or summary measures	p.11		
Main results	16	(a) Give unadjusted estimates and, if applicable, confounder-adjusted estimates and their precision (e.g., 95% confidence interval). Make clear which confounders were adjusted for and why they were included<br>(b) Report category boundaries when continuous variables were categorized<br>(c) If relevant, consider translating estimates of relative risk into absolute risk for a meaningful time period	p.11-12 Table 4&5		
Other analyses	17	Report other analyses done—e.g., analyses of subgroups and interactions, and sensitivity analyses	N/A		
**Discussion**					
Key results	18	Summarise key results with reference to study objectives	p.12-13		
Limitations	19	Discuss limitations of the study, taking into account sources of potential bias or imprecision. Discuss both direction and magnitude of any potential bias	p.14	RECORD 19.1: Discuss the implications of using data that were not created or collected to answer the specific research question(s). Include discussion of misclassification bias, unmeasured confounding, missing data, and changing eligibility over	p.14

For peer review only

				time, as they pertain to the study being reported.	
Interpretation	20	Give a cautious overall interpretation of results considering objectives, limitations, multiplicity of analyses, results from similar studies, and other relevant evidence	p.12-14		
Generalisability	21	Discuss the generalisability (external validity) of the study results	p.12-14		
**Other Information**					
Funding	22	Give the source of funding and the role of the funders for the present study and, if applicable, for the original study on which the present article is based	p.15		
Accessibility of protocol, raw data, and programming code		p.16		RECORD 22.1: Authors should provide information on how to access any supplemental information such as the study protocol, raw data, or programming code.	p.16

*Reference: Benchimol EI, Smeeth L, Guttmann A, Harron K, Moher D, Petersen I, Sørensen HT, von Elm E, Langan SM, the RECORD Working Committee. The REporting of studies Conducted using Observational Routinely-collected health Data (RECORD) Statement. *PLoS Medicine* 2015; in press.

*Checklist is protected under Creative Commons Attribution (CC BY) license.

For peer review only
